# Analyzing classification and feature selection strategies for diabetes prediction across diverse diabetes datasets

**DOI:** 10.3389/frai.2024.1421751

**Published:** 2024-08-21

**Authors:** Jayakumar Kaliappan, I. J. Saravana Kumar, S. Sundaravelan, T. Anesh, R. R. Rithik, Yashbir Singh, Diana V. Vera-Garcia, Yassine Himeur, Wathiq Mansoor, Shadi Atalla, Kathiravan Srinivasan

**Affiliations:** ^1^School of Computer Science and Engineering, Vellore Institute of Technology, Vellore, India; ^2^Radiology, Mayo Clinic, Rochester, MN, United States; ^3^College of Engineering and Information Technology, University of Dubai, Dubai, United Arab Emirates

**Keywords:** machine learning, diabetes prediction, explainable AI, filter-based feature selection, wrapper-based feature selection

## Abstract

**Introduction:**

In the evolving landscape of healthcare and medicine, the merging of extensive medical datasets with the powerful capabilities of machine learning (ML) models presents a significant opportunity for transforming diagnostics, treatments, and patient care.

**Methods:**

This research paper delves into the realm of data-driven healthcare, placing a special focus on identifying the most effective ML models for diabetes prediction and uncovering the critical features that aid in this prediction. The prediction performance is analyzed using a variety of ML models, such as Random Forest (RF), XG Boost (XGB), Linear Regression (LR), Gradient Boosting (GB), and Support VectorMachine (SVM), across numerousmedical datasets. The study of feature importance is conducted using methods including Filter-based, Wrapper-based techniques, and Explainable Artificial Intelligence (Explainable AI). By utilizing Explainable AI techniques, specifically Local Interpretable Model-agnostic Explanations (LIME) and SHapley Additive exPlanations (SHAP), the decision-making process of the models is ensured to be transparent, thereby bolstering trust in AI-driven decisions.

**Results:**

Features identified by RF in Wrapper-based techniques and the Chi-square in Filter-based techniques have been shown to enhance prediction performance. A notable precision and recall values, reaching up to 0.9 is achieved in predicting diabetes.

**Discussion:**

Both approaches are found to assign considerable importance to features like age, family history of diabetes, polyuria, polydipsia, and high blood pressure, which are strongly associated with diabetes. In this age of data-driven healthcare, the research presented here aspires to substantially improve healthcare outcomes.

## 1 Introduction

Diabetes Mellitus, commonly referred to as diabetes, is a widespread global health concern. Historically, it has been prevalent primarily among middle-aged and elderly individuals. However, recent developments, such as technological advancements and the increasing availability of fast food, have contributed to its rising incidence in younger populations. The primary etiology of diabetes is characterized by elevated blood sugar levels due to inefficient insulin utilization within the human body. There are two primary types of diabetes: Type 1, which is characterized by an absolute insulin deficiency with an autoimmune basis, and Type 2, attributed to insulin resistance (Alam et al., 2014). The diagnostic symptoms of diabetes are identified by a plasma glucose concentration exceeding 11.1 mmol/L, accompanied by excessive thirst (polydipsia), unexplained weight loss, and excessive urination (polyuria) (Deshmukh et al., 2015). Diabetes is associated with substantial long-term health risks, including stroke, cardiovascular disease, heart attack, kidney failure, and peripheral arterial disease, as well as complications in blood vessels and nerves (Nathan, 1993; Krasteva et al., 2014). The global population affected by diabetes is projected to more than double by 2030, even under the unlikely scenario that obesity rates remain constant. This anticipated increase is attributed to the effects of urbanization and aging populations. This trend raises significant concern due to the escalating obesity rates worldwide and the substantial role that obesity plays as a risk factor for diabetes (Setacci et al., 2009). Preventative measures for diabetes include the promotion of increased physical activity, adherence to a balanced diet, maintenance of a healthy body mass index, and the cessation of deleterious health behaviors, such as smoking (Suryasa et al., 2021). Individuals with insulin-dependent diabetes and those in the early stages of proliferative retinopathy are two groups that benefit significantly from early detection and prompt treatment of diabetes (Bennett and Knowler, 1984). For a substantial proportion of patients, prompt and efficient diabetes management is found to help prevent vascular complications and slow the further deterioration of already impaired beta-cell function (Ambady and Chamukuttan, 2008).

Within the healthcare industry, big data encompasses complex electronic health datasets that traditional software tools often struggle to manage effectively (Habchi et al., 2023). Healthcare analysis involves the methodical utilization of these datasets to extract valuable insights, support decision-making processes, aid in planning, foster learning, and enable the early prediction and detection of diseases through various models and approaches (Asri et al., 2015; Dash et al., 2019). The continuous progression of machine learning (ML) is an ongoing trend that is closely monitored by the healthcare sector (Patel et al., 2023; Singh et al., 2023). ML principles are instrumental in supporting healthcare professionals and surgeons in saving lives, facilitating early disease identification, improving patient management, enhancing patient engagement in their recovery, and various other applications (Farrelly et al., 2023). Globally, healthcare organizations are leveraging AI-driven solutions and ML models to enhance the delivery of medical services, ultimately facilitating the development of treatments for severe diseases with greater efficiency (Javaid et al., 2022; Dixit et al., 2023).

The proposed contributions of this study include:

- The novelty in this work is the selection of best features from filter and wrapper-based approaches for these four datasets.
- To the best of our knowledge, this is the first time the feature importance study has been applied to these four datasets.
- The comparison between the performance of feature selection and ensemble learning approaches for diabetes prediction is done.
- Explainable techniques SHAP and LIME plots help the clinicians to clearly understand the decision of the ML algorithm and identify the relationships between patient characteristics and diabetes risk.

## 2 Literature survey

### 2.1 Feature extraction-based diabetes prediction

Feature extraction is performed using principal component analysis, followed by the application of resampling filters. Three classifier methods are employed: K-Nearest Neighbors (KNN), Naive Bayes, and Decision Tree. The highest accuracy achieved, 94.4%, is obtained with the Decision Tree classifier (Saru and Subashree, 2019). Building on this approach, Sisodhia also included preprocessing in their analysis, employing classifiers like Naive Bayes, SVM, and Decision Tree. It is found that Naive Bayes exhibits the highest accuracy among the three classifiers (Sisodia and Sisodia, 2018). Extending the use of ML for health diagnostics, Sarwar et al. (2018) developed a model for the early detection of diabetes. This model utilizes a dataset that includes critical features such as the diabetes pedigree function and Body Mass Index (BMI). Several ML algorithms were tested, with SVM and KNN achieving the highest accuracy scores, demonstrating the potential of these techniques in clinical applications.

Similarly, Sharma et al. (2021) selected the Pima Indian Diabetic dataset and applied four ML models for analysis. Among these models, the highest accuracy, recorded at 80.43%, was achieved by the Logistic Regression algorithm, illustrating the effectiveness of this particular model for this dataset. In a targeted effort to reduce diagnostic errors, Mujumdar and Vaidehi (2019) focuses on mitigating false negatives, false positives, and unclassified errors using a structured five-module approach. These modules include data collection, preprocessing, clustering, model development, and evaluation. K-means clustering demonstrated a significant correlation between the “Glucose” and “Age” attributes. Subsequently, Logistic Regression was identified as the most effective model with an accuracy of 96%. Additionally, when pipeline techniques were applied, the AdaBoost classifier achieved an accuracy of 98%, showing the benefits of advanced preprocessing and ensemble methods. Addressing class imbalance, Tasin et al. (2023) centers on predicting insulin characteristics using a semi-supervised model with high gradient boosting. Techniques such as the Synthetic Minority Over-sampling Technique (SMOTE) and Adaptive Synthetic (ADASYN) sampling are utilized. This approach led to an XGBoost classifier reaching the highest accuracy of 81%, an AUC of 0.84, and an F1 score of 0.81, which highlights the utility of gradient boosting in managing skewed data distributions.

Continuing the focus on Type 1 diabetes, Xue et al. (2020) emphasizes the importance of early identification due to the potential long-term damage to vital organs. The study finds SVM to perform best in recognizing early diabetes symptoms, stressing the need for efficient and accurate classifiers in medical prognostics. Further exploring ensemble methods, Vijayan and Anjali (2015) proposes a decision support system using the AdaBoost algorithm with a Decision Stump as the base classifier. When the Decision Tree is employed as the base classifier, AdaBoost achieves an accuracy of 80.72%, showcasing the effectiveness of combining multiple learning algorithms to improve prediction accuracy. On the performance analysis front, Lyngdoh et al. (2021) conducted studies on five supervised ML algorithms to predict diabetes risk. The consistent inclusion of all existing risk variables led to an increase in accuracy, with the KNN classifier achieving up to 76% accuracy. This finding underscores the significance of comprehensive feature selection in developing predictive models (Lyngdoh et al., 2021).

Tripathi and Kumar (2020) contributes to the personalization of patient diagnostics, aligning results closely with clinical outcomes. Utilizing four ML methods, RF was found to surpass other classification algorithms with the highest accuracy of 87.66%, demonstrating its robustness in handling diverse clinical data. In a related study, Shafi and Ansari (2021) investigates the performance of Fasting Plasma Glucose (FPG) and Hemoglobin A1c (HbA1c) as predictive features for diabetes. Multiple ML classifiers and feature elimination techniques were used to achieve favorable results, further emphasizing the role of feature selection in enhancing classification performance. Lastly, Sisodia and Sisodia (2018) and Ahmad et al. (2021) both focus on enhancing the precision in predicting diabetes likelihood. Ahmad et al. (2021) study, using three ML classification methods, found Naive Bayes to be particularly effective with an accuracy of 76.30%. Meanwhile, Sisodia and Sisodia (2018) developed a comprehensive framework aimed at maximizing the precision of diabetes detection using various ML techniques, utilizing the Pima Indian Diabetes dataset from the UCI repository. Together, these studies illustrate ongoing advancements in ML for diabetes prediction, with a focus on accuracy and early detection.

### 2.2 Multi-classification frameworks for diabetes prediction

Abnoosian et al. (2023) present a multi-classification framework using various machine learning models (k-NN, SVM, DT, RF, AdaBoost, and GNB) and a weighted ensemble approach to address challenges such as limited labeled data, frequent missing values, and dataset imbalance. The framework, applied to the Iraqi Patient Dataset of Diabetes, achieves high performance with an average accuracy of 98.87% and AUC of 0.999. Reza et al. (2023) propose an improved non-linear kernel for the SVM model to enhance Type 2 diabetes classification using the PIMA dataset. Their approach addresses missing values and class imbalance, resulting in improved performance metrics such as an accuracy of 85.5% and AUC of 85.5%.

### 2.3 Ensemble learning and hybrid models

Ganie et al. (2023) focus on using five boosting algorithms on the Pima diabetes dataset, with Gradient Boosting achieving the highest accuracy rate of 92.85%. They demonstrate the model's applicability to other diseases with similar indications. Doğru et al. (2023) develop a super ensemble learning model with four base-learners and a meta-learner (SVM), achieving the highest accuracy results for early-stage diabetes risk prediction (99.6%), PIMA (92%), and diabetes 130-US hospitals (98%) datasets. Zhou et al. (2023) propose a diabetes prediction model using Boruta feature selection and ensemble learning, validated on the PIMA Indian diabetes dataset, achieving an accuracy rate of 98%.

### 2.4 Deep learning approaches

El-Bashbishy and El-Bakry (2024) present a deep learning-based model using a DNN-based multi-layer perceptron (MLP) algorithm for early diabetes prediction, achieving a high accuracy rate of 99.8% on the Mansoura University Children's Hospital Diabetes (MUCHD) dataset.

### 2.5 Comparative evaluations and hybrid techniques

Saxena et al. (2023) provide a comparative evaluation of classical and ensemble machine learning models on the PIMA Indian diabetes dataset and an early-stage diabetes risk prediction dataset. The superlearner model provides the best accuracy of 86% for PIMA and 97% for the early-stage diabetes risk prediction dataset. Tasin et al. (2023) develop an automatic diabetes prediction system using various machine learning techniques and a private dataset of female patients in Bangladesh. The XGBoost classifier with the ADASYN approach provides the best results with 81% accuracy. Tripathi et al. (2023) analyze various machine learning algorithms and classifiers on the PIMA diabetes dataset, using soft voting ensemble techniques to achieve the highest accuracy.

### 2.6 Innovative and nature-inspired algorithms

Jain and Singhal (2024) use nature-inspired metaheuristic algorithms like the Bat Algorithm and Voting classifier with Smote for diabetes prediction, achieving a maximum accuracy of 98%. Alnowaiser (2024) propose an automated method for predicting diabetes using KNN imputer and a Tri-ensemble voting classifier, achieving an accuracy of 97.49%. Shimpi et al. (2024) present an analytical model using optimized SVM, KNN, and Random Forest with decision-level fusion and Particle Swarm Optimization, achieving a prediction rate of 94.27%.

### 2.7 Data mining and model fusion

Rastogi and Bansal (2023) propose a diabetes prediction model using data mining techniques and four classifiers, with Logistic Regression achieving the highest accuracy of 82.46%. Zohair et al. (2024) develop a hybrid model using ANN, AdaBoost, and RF with Logistic Regression for binary and multiclass classification of diabetes, achieving 97% accuracy for binary classification and 99% for multiclass. Table 1 presents a comparison of various studies on diabetes prediction based on ML.

**Table 1:** Comparison of various studies on diabetes prediction.

**Reference**	**Model used**	**Data types or dataset**	**Application**	**Best performance value**	**Limitation**
Abnoosian et al. (2023)	k-NN, SVM, DT, RF, AdaBoost, GNB	Iraqi Patient Dataset of Diabetes	Diabetes prediction in three classes	Accuracy: 0.9887, Precision: 0.9861, Recall: 0.9792, F1-score: 0.9851, AUC: 0.999	Limited labeled data, frequent missing values, dataset imbalance
Reza et al. (2023)	SVM with improved non-linear kernel	PIMA dataset	Type 2 diabetes classification	ACC: 85.5, Recall: 87.0, Precision: 83.4, F1 score: 85.2, AUC: 85.5	Kernel function choice impacts performance
Ganie et al. (2023)	Gradient Boosting	Pima diabetes dataset (UCI repository)	Early diabetes prediction	Accuracy: 92.85%	Limited to Pima dataset, potential overfitting
Saxena et al. (2023)	Superlearner model	PIMA Indian diabetes dataset, early-stage diabetes risk prediction dataset	Diabetes risk prediction	Accuracy: 86% (PIMA), 97% (risk prediction dataset)	Performance varies with dataset
Tasin et al. (2023)	XGBoost with ADASYN	Pima Indian diabetes dataset, private dataset of female Bangladeshi patients	Diabetes prediction	Accuracy: 81%, F1 score: 0.81, AUC: 0.84	Dataset-specific performance, need for domain adaptation
Tripathi et al. (2023)	Various ML algorithms and classifiers	Standardized PIMA diabetes data	Diabetes prediction	Highest accuracy achieved using soft voting ensemble techniques	Limited to standardized PIMA data
Doğru et al. (2023)	Super learner model (logistic regression, DT, RF, gradient boosting, SVM)	Early-stage diabetes risk prediction, PIMA, diabetes 130-US hospitals dataset	Early diagnosis of diabetes mellitus	Accuracy: 99.6% (risk prediction), 92% (PIMA), 98% (130-US hospitals)	Dataset-specific performance, complexity of super learner model
Rastogi and Bansal (2023)	RF, SVM, Logistic Regression, Naive Bayes	Kaggle dataset	Diabetes prediction	Accuracy: 82.46% (Logistic Regression)	Lower accuracy compared to other studies
Zhou et al. (2023)	Boruta feature selection, K-Means++, stacking ensemble	PIMA Indian diabetes dataset	Early detection of diabetes	Accuracy: 98%	Limited to PIMA dataset
El-Bashbishy and El-Bakry (2024)	DNN-based MLP algorithm	MUCHD dataset	Early diabetes prediction	Accuracy: 99.8%	Dataset-specific performance, potential for overfitting
Modak and Jha (2024)	Logistic Regression, SVM, Näve Bayes, Random Forest, XGBoost, LightGBM, CatBoost, Adaboost, Bagging	Kaggle dataset	Diabetes prediction	Accuracy: 95.4% (CatBoost), AUC-ROC: 0.99	Limited to Kaggle dataset, ensemble methods complexity
Zambrana et al. (2024)	Ridge Classifier, Random Forest, Decision Tree	Two diabetes datasets	Diabetes classification	Accuracy: 95% (Random Forest, Decision Tree)	Limited dataset, potential overfitting
Wee et al. (2024)	Machine learning and deep learning models	Various datasets	Diabetes identification/classification	Accuracy: 86.7% (deep learning), 80.6% (machine learning)	Limited dataset availability, "black-box" nature of deep learning
Jain and Singhal (2024)	Ant Colony Optimization, Bat Algorithm, Cuttlefish Algorithm, Elephant Herd Optimization, Artificial Bee Algorithm	Specific dataset	Diabetes prediction	Accuracy: 98% (Voting classifier with Smote and Bat Algorithm)	Dataset-specific performance, complexity of nature-inspired algorithms
Shimpi et al. (2024)	SVM, KNN, Random Forest, Particle Swarm Optimization (PSO)	Indian Pima diabetes dataset	Diabetes detection	Accuracy: 94.27% (Hybrid classifiers)	Tedious hyper-parameter tuning, dataset-specific performance
Alnowaiser (2024)	KNN imputer, Tri-ensemble voting classifier	Various datasets	Diabetes prediction	Accuracy: 97.49%, Precision: 98.16%, Recall: 99.35%, F1 score: 98.84%	Handling of missing data, model complexity
Zohair et al. (2024)	ANN, AdaBoost, RF, Logistic Regression	Various datasets	Diabetes classification	Accuracy: 97% (binary), 99% (multiclass)	Dataset-specific performance, complexity of hybrid model
Talari et al. (2024)	SMO, SMOTE, Bagging Decision Trees	Pima Indian Diabetes (PID) dataset	Diabetes prediction	Accuracy: 99.07%, Runtime: 0.1 ms	Limited to PID dataset, potential overfitting

## 3 Proposed methodology

**Figure 1:**
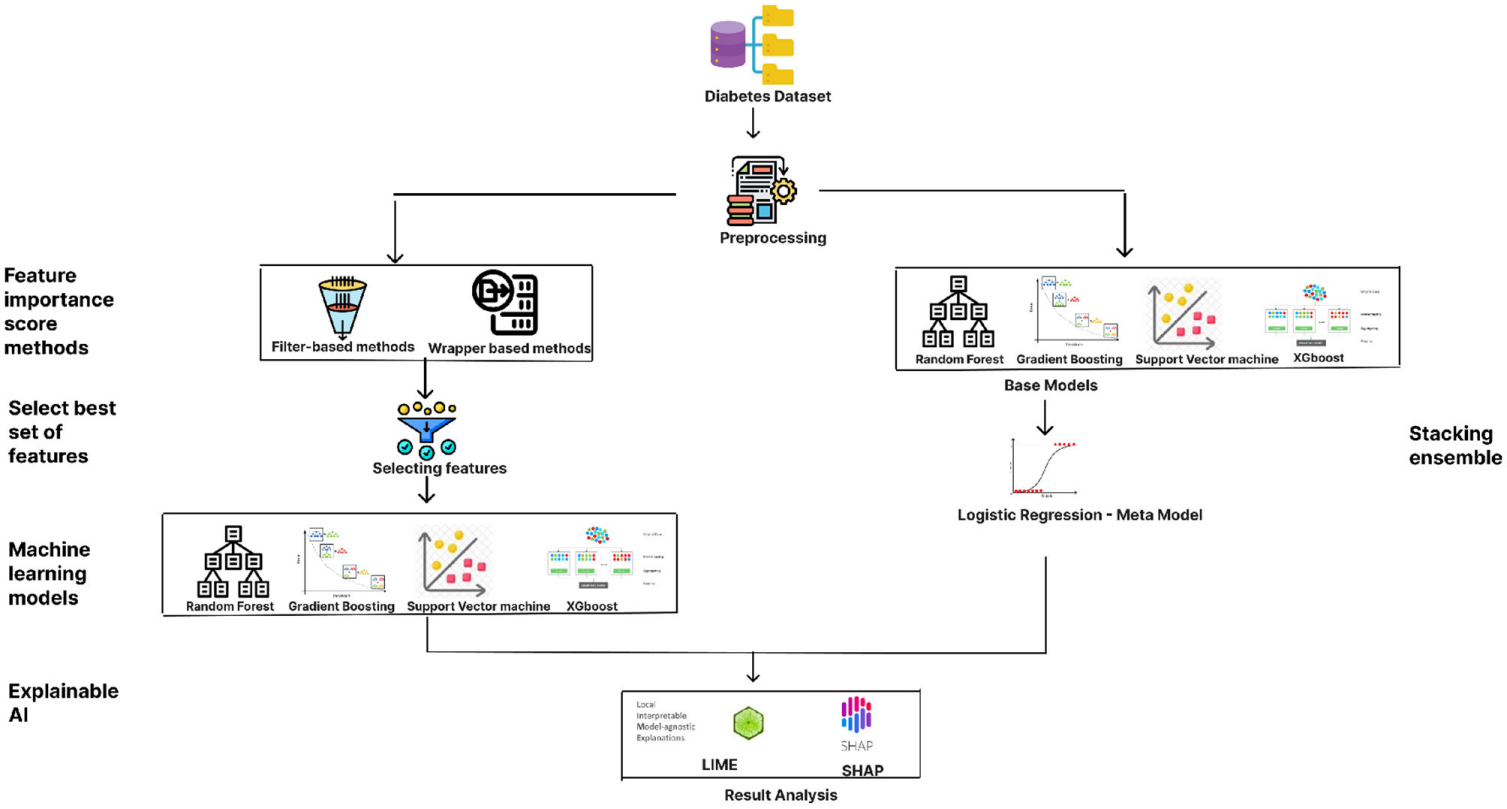
Flowchart of the proposed methodology.

The proposed methodology for predicting diabetes, as illustrated in Figure 1, begins with data preprocessing, an essential step to ensure clean and usable data. The preprocessing stage is followed by the application of two categories of feature-importance scoring methods: filter-based techniques and wrapper methods. The filter-based methods implemented include Chi-square, Fisher's Score, analysis of missing values, and Information Gain, while the wrapper methods incorporate models such as Random Forest (RF), XGBoost Classifier, Gradient Boosting (GB) Classifier, Support Vector Machine (SVM), and Logistic Regression (LR).

The best set of features resulted from the filter and wrapper based methods is chosen. The dataset's accuracy is evaluated by considering both the full set of features and the subset of key features identified through feature importance scoring. The ML models employed for performance evaluation include XGBoost, Gradient Boosting, SVM, and Random Forest. Next study on the method uses an ensemble method, the stacking classifier, is utilized to compare performance, with the aforementioned models serving as base models and Logistic Regression as the meta-model. Finally, to interpret and validate the results produced by the ML algorithms, explainable AI techniques such as Local Interpretable Model-agnostic Explanations (LIME) and SHapley Additive exPlanations (SHAP) are applied. Performance metrics such as accuracy score, precision, recall, and F1 score are measured for both the full feature set and the key features to comprehensively understand their impact on diabetes prediction.

### 3.1 Feature importance selection

Feature selection, also known as variable selection, attribute selection, or variable subset selection, is a critical phase in ML and data analysis. From the entire set of features in the dataset, a subset of relevant features (predictors, characteristics, or input variables) is chosen to build a model. The objective of feature selection is to enhance interpretability, reduce computational complexity, and improve model performance by focusing on the most informative and discriminative characteristics (Wei et al., 2020).

### 3.2 Wrapper based feature selection

Wrapper-based feature selection is a ML technique that approaches the process of selecting subsets of features as a search problem. This method involves training and evaluating the effectiveness of ML models with various feature subsets to identify the one that provides the best prediction performance. Wrapper methods evaluate feature subsets by applying a specific ML algorithm as a “wrapper” around the feature selection process.

#### 3.2.1 Random forest

Random Forest is a technique where a multitude of decision trees are constructed during training using ensemble ML, and their predictions are then aggregated to produce a final prediction. It is widely applied in problems involving both regression and classification due to its accuracy and robustness.

Formula for Random Forest (feature importance using Gini impurity):

In Random Forest, feature importance is often calculated based on the average Gini impurity reduction attributed to each feature over all the trees in the forest. Let imp(*F*) represent the importance score for feature (*F*) and it is calculated using Equation 1.


(1) $$\begin{array}{l} \text{imp}\left(F\right)=\frac{1}{N_{\text{trees}}}\overset{N_{\text{trees}}}{\underset{i=1}{\sum }}\text{imp}\left(F,i\right) \end{array}$$

where:

- *N*_trees_ is the total number of trees in the Random Forest.
- imp(*F, i*) is the Gini impurity reduction for feature (*F*) in the *i*th tree.

The higher the importance score (*F*), the more relevant the feature is in contributing to the predictive performance of the Random Forest model.

#### 3.2.2 Gradient boosting

Gradient Boosting is an ensemble method in ML that systematically combines the predictions of several weak learners, typically decision trees, so that each new learner addresses the shortcomings of its predecessor. This approach involves employing gradient descent to minimize a loss function during training, thereby creating a powerful predictive model.

Formula for Gradient Boosting (feature importance using gain):

In Gradient Boosting, feature importance is often computed based on the average gain (or improvement) in the loss function attributed to each feature over all the boosting iterations. Let gain(*F*) represent the average gain for a feature (*F*) calculated using Equation 2.


(2) $$\begin{array}{l} \text{gain}\left(F\right)=\frac{1}{M}\overset{M}{\underset{m=1}{\sum }}\overset{N_{m}}{\underset{i=1}{\sum }}\text{Gain}\left(F,i,m\right) \end{array}$$

where:

- *M* is the total number of boosting iterations.
- *N*_*m*_ is the number of samples at iteration *m*.
- Gain(*F, i, m*) is the gain for feature (*F*) at iteration *m* for sample *i*.

The higher the average gain gain(*F*), the more important the feature is in contributing to the model's predictive performance.

#### 3.2.3 XGBoost feature selection

Gradient boosting is executed effectively and in a scalable manner by XGBoost, an ensemble ML technique. XGBoost progressively constructs a series of weak learners, typically decision trees, and employs gradient descent to minimize a loss function. This approach contributes to the high performance and widespread use of XGBoost in various ML tasks.

XGBoost calculates feature importance based on the average gain (or improvement) in the loss function attributed to each feature over all the boosting iterations. Let gain(*F*) represent the average gain for feature (*F*) and it is calculated using Equation 3.


(3) $$\begin{array}{l} \text{gain}\left(F\right)=\frac{1}{M}\overset{M}{\underset{m=1}{\sum }}\text{Gain}\left(F,m\right) \end{array}$$

where:

- *M* is the total number of boosting iterations.
- Gain(*F, m*) is the gain for feature (*F*) at iteration (*m*).

The higher the average gain gain(*F*), the more important the feature is in contributing to the model's predictive performance.

#### 3.2.4 Support vector machine

SVM is a proficient supervised ML technique used to address regression and classification problems. They identify the optimal decision boundary (hyperplane) to separate different classes or predict values by optimizing the distance between data points and the hyperplane.

In SVM, the importance of features can be assessed using the absolute values of the weights assigned to each feature in the optimal hyperplane. Let *w*_*i*_ represent the weight for feature (*i*), and imp(*F*_*i*_) represent the importance of feature (*i*) found using Equation 4.


(4) $$\begin{array}{l} \text{imp}\left(F_{i}\right)=|w_{i}| \end{array}$$

The higher the absolute weight |*w*_*i*_|, the more important the feature is in defining the hyperplane and contributing to the SVM's predictive performance.

#### 3.2.5 Linear regression

Linear regression is a basic supervised ML technique for regression applications. It illustrates the relationship between a dependent variable (y) and one or more independent variables (x) by fitting a linear equation to the observed data. To minimize the discrepancy between actual and anticipated values, the best-fitting line (or hyperplane in higher dimensions) must be found.

In Linear Regression, feature importance can be assessed using the absolute values of the coefficients assigned to each feature in the linear equation. Let *β*_*i*_ represent the coefficient for feature (*i*), and imp(*F*_*i*_) represent the importance of feature (*i*). Equation 5 shows how imp(*F*_*i*_) is determined.


(5) $$\begin{array}{l} \text{imp}\left(F_{i}\right)=|\beta_{i}| \end{array}$$

The higher the absolute coefficient |*β*_*i*_|, the more important the feature is in determining the outcome and contributing to the predictive performance of the Linear Regression model.

### 3.3 Filter based model

Filter-based feature selection is a ML feature selection method that evaluates the importance of each feature independently, using statistical or mathematical measurements, without reference to any specific ML model. In this approach, features are ranked or scored based on their characteristics, and a subset of the most informative features is selected for further use in model training. This process is conducted as a preprocessing step before training a ML model, thereby forming an integral part of the feature selection process.

#### 3.3.1 Chi-square

The Chi-Square test, a statistical technique for feature selection, is particularly useful for categorical data. It assesses whether there is a dependence or relationship between a categorical feature and the target variable by comparing the observed frequency of each category with the expected frequency under the assumption of independence.

For a given feature *F* with categories *C*_1_, *C*_2_, …, *C*_*k*_ and target variable (*T*), the Chi-Square statistic χ^2^ for that feature can be calculated as given in Equation 6:


(6) $$\begin{array}{l} \chi^{2}=\overset{k}{\underset{i=1}{\sum }}\frac{{\left(O_{i}-E_{i}\right)}^{2}}{E_{i}} \end{array}$$

where:

- *O*_*i*_ is the observed frequency of category *C*_*i*_ in the feature (*F*).
- *E*_*i*_ is the expected frequency of category *C*_*i*_ in the feature (*F*), assuming independence between (*F*) and (*T*).

The higher the χ^2^ value, the more important the feature is in relation to the target variable.

#### 3.3.2 Fisher's score

Fisher's Score is recognized as a statistical technique pivotal for feature selection within the domain of ML. This method evaluates the discriminatory power of a feature by analyzing the ratio of the variance within each class to the disparity in mean values across different classes.

For a given feature (*F*) with (*K*) categories and a target variable (*T*), Fisher's Score *F*(*F*) is calculated as per the Equation 7:


(7) $$\begin{array}{l} F\left(F\right)=\frac{\text{mean difference between classes}\left(F\right)}{\text{variance within classes}\left(F\right)} \end{array}$$

where:

- Mean difference between classes (*F*) measures the difference in means of feature (*F*) across different classes.
- Variance within classes (*F*) measures the variance of feature (*F*) within each class.

A higher Fisher's Score indicates a more discriminative feature that can be considered important for selection.

#### 3.3.3 Missing value

Before the application of ML models, it is essential to identify and address instances or features containing missing data. The management of these missing values is conducted using a filter-based method. This approach determines the strategies for handling or imputing missing values, guided by statistical considerations or factors specific to the dataset.

The mean imputation method mentioned in Equation 8 replaces missing values with the mean of the non-missing values for a specific feature (*F*):


(8) $$\begin{array}{l} \text{MeanImputation}\left(F\right)=\frac{1}{N}\overset{N}{\underset{i=1}{\sum }}{\text{ValidValues}}_{i} \end{array}$$

where:

- *N* is the number of non-missing (valid) values for feature (*F*).
- ValidValues_*i*_ represents each valid value of feature (*F*).

The median imputation method given in Equation 9 replaces missing values with the median of the non-missing values for a specific feature (*F*):


(9) $$\begin{array}{l} \text{MedianImputation}\left(F\right)=\text{Median}\left({\text{ValidValues}}_{i}\right) \end{array}$$

where:

- Median(ValidValues_*i*_) is the median of the non-missing (valid) values for feature (*F*).

The effectiveness of handling missing values in data relies on statistical measures derived directly from the dataset, characterizing these imputation methods as filter-based. It is vital to acknowledge that imputation using mean or median values can introduce biases. Consequently, such methods must be employed with caution, considering their impact on the integrity of the entire dataset and implications for future research.

#### 3.3.4 Information gain

Information Gain stands as a crucial statistical metric utilized in the realm of ML for the purpose of feature selection. This metric quantifies the extent of knowledge obtained about the target variable through awareness of a feature's value. A higher Information Gain indicates that the feature significantly contributes to predicting the target variable, underscoring its utility in the model.

Information Gain IG(*F*) for a feature (*F*) is calculated using entropy, a measure of the amount of uncertainty in a set of data as shown in Equation 10. Let *H*(*T*) represent the entropy of the target variable, and *H*(*T*|*F*) represent the conditional entropy of the target variable given feature (*F*).


(10) $$\begin{array}{l} \text{IG}\left(F\right)=H\left(T\right)-H\left(T|F\right) \end{array}$$

The entropy of the target variable *H*(*T*) and the conditional entropy *H*(*T*|*F*) are given in Equation 11 and Equation 12


(11) $$\begin{array}{l} H\left(T\right)=-\overset{c}{\underset{i=1}{\sum }}p_{i}\log_{2}\left(p_{i}\right) \end{array}$$

(12) $$\begin{array}{l} H\left(T|F\right)=\overset{k}{\underset{j=1}{\sum }}\frac{\left|F_{j}\right|}{\left|T\right|}H\left(T_{j}\right) \end{array}$$

where:

- *c* is the number of classes in the target variable.
- *p*_*i*_ is the proportion of samples in class (*i*).
- *k* is the number of unique values in the feature (*F*).
- |*F*_*j*_| is the number of samples with a value (*j*) in feature (*F*).
- |*T*| is the total number of samples.
- *H*(*T*_*j*_) is the entropy of the target variable for samples with a value (*j*) in feature (*F*).

### 3.4 Feature selection in ML

In the context of feature selection for ML, the relevance and utility of a feature in predicting the target variable are directly proportional to its Information Gain. A larger Information Gain signifies a more pertinent and beneficial feature in forecasting outcomes.

## 4 Classification using ML models

Classification in ML involves training an algorithm or model, referred to as a classifier, to categorize or label input data points based on identifying patterns and attributes within the data. The primary goal of a classifier is to establish a mapping from input attributes to predetermined categories or classes, which is then utilized to make predictions on new, unseen data points.

### 4.1 Random forest classifier

The Random Forest classifier, an ensemble ML technique, leverages the collective strength of multiple decision trees to yield more accurate predictions or classifications. Introduced by Leo Breiman and Adele Cutler, this method has gained widespread application in various data science and ML tasks. Notably, the accuracy of the Random Forest approach improves with the expansion of the dataset. Furthermore, an increase in data with a similar proportion of cases also enhances accuracy. It has been observed that combining the Genetic Algorithm with the Random Forest method results in higher accuracy for diabetes mellitus datasets compared to other variants of the Random Forest method. The prediction formula is given in Equation 13.


(13) $$\begin{array}{l} P=\frac{1}{n}\overset{n}{\underset{i=1}{\sum }}C_{i} \end{array}$$

where:

- *n* = number of trees in the random forest,
- *C*_*i*_ = represent the regression prediction of the *i*-th decision tree.

#### 4.1.1 Gradient boosting classifier

Gradient Boosting represents an ensemble learning approach, wherein multiple weak learners, such as decision trees, are trained in succession to develop an additive model. Each successive learner is designed to rectify the errors made by its predecessors. The final prediction formula is given in Equation 14.


(14) $$\begin{array}{l} F\left(x\right)=\overset{M}{\underset{m=1}{\sum }}\alpha_{m}f_{m}\left(x\right) \end{array}$$

where:

- *F*(*x*) is the final prediction for input data *x*,
- *M* is the total number of learners in the ensemble,
- *f*_*m*_(*x*) is a prediction of the *m*-th weak learner,
- α_*m*_ is the learning rate for the *m*-th weak learner.

#### 4.1.2 Support vector machine

A Support Vector Machine is a ML model designed to optimize the margin between distinct classes within a dataset by identifying the optimal hyperplane as per the Equation 15. This approach is effective for both classification and regression tasks.


(15) $$\begin{array}{l} f\left(x\right)=\text{sign}\left(w\cdot x+b\right) \end{array}$$

where:

- *w*·*x* is the dot product of the weight factor *w* and feature vector *x*,
- *b* is the bias term,
- sign(.) is the sign function which returns +1 or -1.

#### 4.1.3 XGBoost classifier

XGBoost, or Extreme Gradient Boosting, augments the gradient boosting framework with techniques such as regularization, parallel computing, and tree-pruning. The prediction formula is given in the Equation 16.


(16) $$\begin{array}{l} P\left(x\right)=\overset{M}{\underset{m=1}{\sum }}\gamma F_{m}\left(x\right) \end{array}$$

where:

- *F*_*m*_(*x*) represents the prediction made by the *m*-th decision tree,
- γ is the learning rate.

### 4.2 Ensemble approach

In this study, the ensemble method employed for performance comparison is the stacking classifier, which enhances predictive accuracy by combining multiple ML models. The stacking ensemble approach involves using several base models to make individual predictions, and then a meta-model to integrate these predictions into a final output. Specifically, the base models utilized in this methodology are Random Forest, Gradient Boosting, Support Vector Machine, and XGBoost. These models were selected for their diverse algorithmic approaches and strong performance in classification tasks. The meta-model employed is Logistic Regression, chosen for its simplicity and effectiveness in aggregating the outputs of the base models. By leveraging the strengths of each base model and the meta-model, the stacking ensemble aims to improve the overall prediction performance compared to using a single model.

### 4.3 Blackbox evaluation

The advancement of ML algorithms has significantly enhanced predictive capabilities, often at the expense of interpretability. Modern models, while capable of making highly accurate predictions, frequently present challenges in understanding their decision-making processes. To mitigate this, Explainable AI (Artificial Intelligence) has been developed to create models that are not only high-performing but also more interpretable.

Two prominent methods employed for the evaluation and explanation of ML model performance are LIME and SHAP:

**LIME (Local Interpretable Model-Agnostic Explanations)**: LIME focuses on elucidating the outputs of classifiers or regressors. This is accomplished by approximating a complex model's behavior with a simpler, more interpretable model, enhancing human understanding. LIME's effectiveness lies in its ability to offer insights into a model's decision-making process for specific predictions. It provides the option to choose between two interpretable classifiers and has demonstrated considerable generalizability in various applications.

**SHAP (SHapley Additive exPlanations)**: SHAP extends this explanatory capability by offering a feature importance score for each attribute in every prediction. It quantifies the contribution of each feature to a particular prediction, thereby allowing a deeper insight into the model's functionality. SHAP is distinguished by its introduction of a new class of cumulative feature significance indicators, which contribute to a more comprehensive assessment of feature importance. The methodology also uncovers various desirable attributes for explaining model predictions, adding to its robustness.

In summary, LIME and SHAP are indispensable tools in the realm of ML for model evaluation and interpretation. While LIME facilitates the creation of local, interpretable models that approximate the behavior of more complex systems, SHAP provides detailed insights into how each feature influences individual predictions. These approaches enhance the transparency and trustworthiness of AI models, playing a crucial role in their application across critical sectors such as healthcare, finance, and autonomous systems.

### 4.4 Performance metrics

The binary classification of having diabetes produces four outcomes: True Positive (TP), True Negative (TN), False Positive (FP), and False Negative (FN).

- **True Positive (TP)**: Correct positive prediction
- **True Negative (TN)**: Correct negative prediction
- **False Positive (FP)**: Incorrect positive prediction
- **False Negative (FN)**: Incorrect negative prediction

#### 4.4.1 Accuracy

Model prediction accuracy is defined as the ratio of correctly identified samples to total samples by the model and it is given in Equation 17.


(17) $$\begin{array}{l} \text{Accuracy}=\frac{TP+TN}{TP+TN+FP+FN} \end{array}$$

#### 4.4.2 Precision

The precision formula given in Equation 18 shows the ratio of successfully classified positive values to all anticipated positive samples serves as a proxy for the model's accuracy.


(18) $$\begin{array}{l} \text{Precision}=\frac{TP}{TP+FP} \end{array}$$

#### 4.4.3 Recall

The ratio of accurately predicted positive samples to all positive samples is known as the recall of a model and it is given in Equation 19.


(19) $$\begin{array}{l} \text{Recall}=\frac{TP}{TP+FN} \end{array}$$

#### 4.4.4 F1-Score

The F1 score of the model determines the harmonic mean of Precision and Recall found using Equation 20.


(20) $$\begin{array}{l} \text{F1-Score}=\frac{2\cdot \text{Precision}\cdot \text{Recall}}{\text{Precision}+\text{Recall}} \end{array}$$

## 5 Results and discussion

### 5.1 Dataset description

Four diabetes datasets were used in our work and all of them have a binary predictor variable. The dataset-1 was created in the year 2020, it has 17 attributes. Dataset-2 includes demographic data also, it contains 9 attributes. The Dataset-3 holds 17 attributes used in the early diagnosis of diabetes. Dataset-4 contains 8 attributes specific to the female. The details of the datasets are given in Table 2.

**Table 2:** Dataset description.

**Dataset name**	**List of features**	**No. of attributes**	**No. of train records**	**No. of test records**
Dataset-1	Age, Gender, Family Diabetes, High BP, Physically Active, BMI, Smoking, Alcohol, Sleep, Soundsleep, Regular medicine, Junk food, Stress, Bp level, Pregnancies, Pdiabetes, Urination Freq, Diabetic^a^	17	760	191
Dataset-2	Gender, Age, Hypertension, Heart disease, Smoking history, Bmi, HbA1c level, Blood glucose level, Diabetes^b^	8	1199	300
Dataset-3	Alopecia, Delayed healing, Visual blurring, Polydipsia, Obesity, Age, Polyphagia, Muscle stiffness, Gender, Itching, Partial paresis, Polyuria, Sudden weight loss, Genital thrush, Irritability, Weakness, Class^c^	16	416	104
Dataset-4	BMI, Age, Pregnancies, Insulin, Glucose, Diabetes Pedigree Function, Blood pressure, Skin thickness, Outcome^d^	8	2214	554

^a^https://www.kaggle.com/datasets/tigganeha4/diabetes-dataset-2019.

^b^https://www.kaggle.com/datasets/iammustafatz/diabetes-prediction-dataset.

^c^https://www.kaggle.com/datasets/andrewmvd/early-diabetes-classification.

^d^https://www.kaggle.com/datasets/mathchi/diabetes-data-set.

### 5.2 Box plot diagrams

Box plot diagrams provide a visual summary of multiple datasets, enabling a clear comparison of distributional characteristics across different groups or conditions. Each box plot in the figures represents the statistical distribution of features within the respective dataset, emphasizing the median, quartiles, and potential outliers. Supplementary Figures 1, 2 illustrate the distribution of features within the four datsets used in the experical evaluation. The interquartile range (IQR), which represents the middle 50% of the data, is highlighted, alongside any potential outliers that are marked as individual points beyond the whiskers. These box plots are essential tools for preliminary data analysis, providing a quick visual understanding of the data's structure, and highlighting differences between datasets that might warrant further investigation.

### 5.3 Correlation matrix

**Figure 2:**
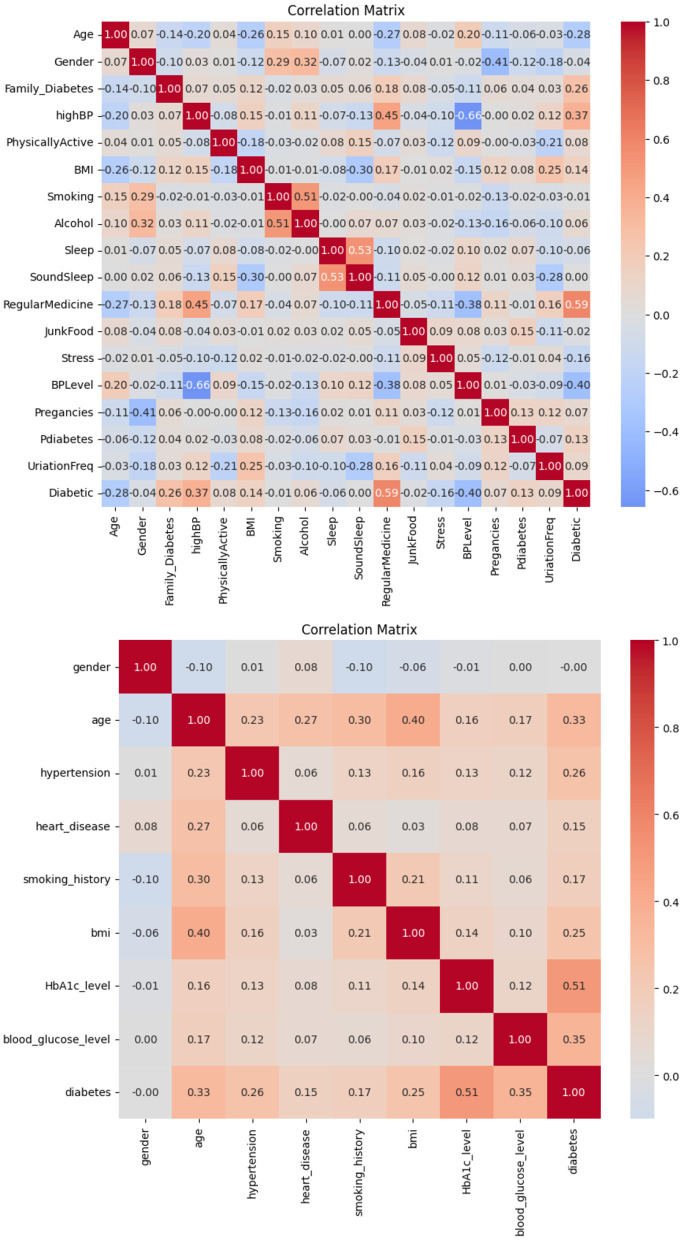
Correlation matrices obtained on **(top)** Dataset-1, and **(bottom)** Dataset-2.

The correlation observed in Dataset-1 and Dataset-2, as illustrated in Figure 2, suggests a significant positive association between regular medication, high blood pressure, and family history of diabetes with the incidence of diabetes. Conversely, blood pressure level and age demonstrate a notable negative correlation. Consequently, it is inferred that these factors play a pivotal role in predicting the likelihood of diabetes in patients.

**Figure 3:**
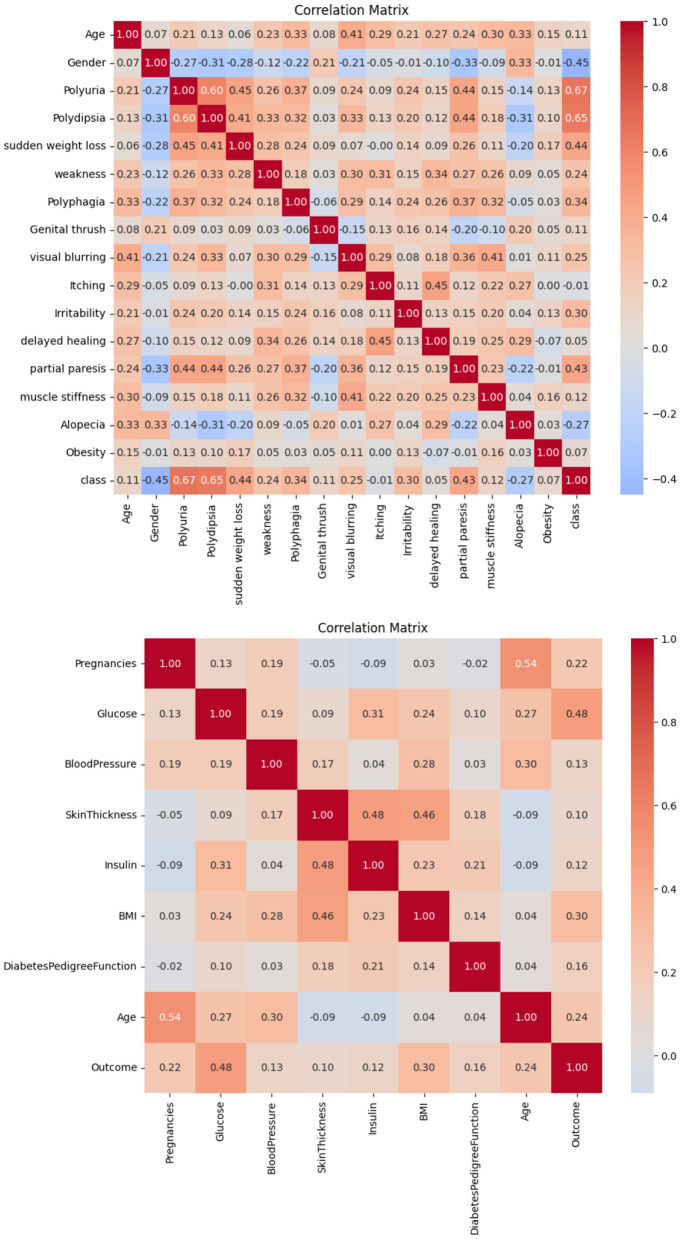
Correlation matrices obtained on **(top)** Dataset-3 and **(bottom)** Dataset-4.

Based on the information in Figure 3, it is possible to conclude that BMI and glucose have a strong positive connection with diabetes, suggesting that these variables have a major impact on predicting a patient's likelihood of having diabetes. On the other hand, no feature in this dataset exhibits a negative connection with diabetes.

### 5.4 Feature importance scores using wrapper based methods

Feature importance scores are found using wrapper-based methods with Random forest (Supplementary Figure 3), XGBoost (Supplementary Figure 4), Gradient Boosting (Supplementary Figure 5), SVM (Supplementary Figure 6), and Linear regression (Supplementary Figure 7) classifiers as wrappers.

In the analysis of Dataset 1, several wrapper-based techniques were utilized to evaluate the feature importance scores of various attributes. Notably, among the assessed features, regular medication, age, and BMI are found to exhibit the highest levels of feature importance. In contrast, attributes such as gender, smoking, and consumption of junk food demonstrate the lowest levels of feature importance within the dataset.

Various wrapper-based techniques were employed to assess the significance of attributes within Dataset 2. It is observed that HbA1c levels, blood glucose levels, and BMI emerge as the attributes with the highest feature importance scores in this dataset. Conversely, gender and smoking history are identified as having the lowest levels of importance.

A range of wrapper-based techniques was applied to determine the feature importance scores of attributes within Dataset 3. It is noteworthy that polyuria, polydipsia, and gender are identified as the attributes with the highest levels of feature importance in this dataset. In contrast, obesity, weakness, and genital thrush are among the features that exhibit the lowest levels of importance.

Several wrapper-based techniques were utilized to evaluate the feature importance scores of attributes within Dataset 4. It is observed that glucose, BMI, and the Diabetes Pedigree Function are the attributes with the highest levels of feature importance in this dataset. Conversely, attributes such as pregnancies, blood pressure, skin thickness, insulin, and age are found to exhibit the lowest levels of importance.

### 5.5 Important features using filter based methods

Filter-based methods were utilized to determine the most significant features in Dataset 1. As per the results documented in Table 3, age, family history of diabetes, and high blood pressure are identified as pivotal factors within the dataset according to these filter-based methods.

**Table 3:** Dataset-1 top 7 features using filter based methods.

**Methods**	**Feature 1**	**Feature 2**	**Feature 3**	**Feature 4**	**Feature 5**	**Feature 6**	**Feature 7**
Chi-Square	Age	Family_Diabetes	HighBP	BMI	Regular medicine	BPLevel	Pdiabetes
Fisher's score	Age	Family_Diabetes	HighBP	BMI	Regular medicine	Stress	BPLevel
Missing value	Age	Family_Diabetes	HighBP	Regular medicine	Stress	BPLevel	Pdiabetes
Information gain	Regular medicine	Age	BPLevel	HighBP	BMI	Stress	Pregnancies

Filter-based methods were utilized to identify the most important features in Dataset 2. The findings, detailed in Table 4, indicate that while most features exhibit similar levels of importance, age, hypertension, and BMI are distinguished as notably pivotal factors in the dataset, as per these filter-based methods.

**Table 4:** Dataset-2 top 5 features using filter based methods.

**Methods**	**Feature 1**	**Feature 2**	**Feature 3**	**Feature 4**	**Feature 5**
Chi-Square	Age	Hypertension	BMI	HbA1c_level	blood_glucose_level
Fisher's score	Age	Hypertension	BMI	HbA1c_level	blood_glucose_level
Missing value	Age	Hypertension	BMI	HbA1c_level	blood_glucose_level
Information gain	BMI	HbA1c_level	blood_glucose_level	Age	Hypertension

The principal elements in Dataset 3 were identified using filter-based techniques. According to the results detailed in Table 5, the significant factors within the dataset, as determined by these filter-based approaches, are gender, polyuria, and polydipsia.

**Table 5:** Dataset-3 top 7 features using filter based methods.

**Methods**	**Feature 1**	**Feature 2**	**Feature 3**	**Feature 4**	**Feature 5**	**Feature 6**	**Feature 7**
Chi-Square	Age	Gender	Polyuria	Polydipsia	Sudden weight loss	Irritability	Partial paresis
Fisher's score	Gender	Polyuria	Polydipsia	Sudden weight loss	polyphagia	Irritability	Partial paresis
Missing value	Gender	Polyuria	Polydipsia	Sudden weight loss	Irritability	Partial paresis	Alopecia
Information gain	Polyuria	Polydipsia	Age	Gender	Sudden weight loss	Partial paresis	Polyphagia

The primary elements in Dataset-4 were discerned through the application of filter-based techniques. As indicated by the results in Table 6, the significant factors within this dataset, as identified by these filter-based approaches, include pregnancies, glucose, and skin thickness.

**Table 6:** Dataset-4 top 5 features using filter-based methods.

**Methods**	**Feature 1**	**Feature 2**	**Feature 3**	**Feature 4**	**Feature 5**
Chi-Square	Pregnancies	Glucose	Skin Thickness	Insulin	BMI
Fischer's score	Pregnancies	Glucose	Blood pressure	Insulin	BMI
Missing value	Pregnancies	Glucose	Skin thickness	BMI	Diabetes pedigree function
Information gain	Diabetes pedigree function	BMI	Glucose	Insulin	Age

The next step involves identifying the best features recommended by filter and wrapper approaches that are common to each dataset. Table 7 presents the best sets of features selected for the four datasets.

**Table 7:** Best set of features given by filter and wrapper-based approaches.

**Dataset**	**Best set of features**
1	Regular medicine, Age, BMI, Sound sleep, BP level, Stress, and Physical activity
2	Age, Hypertension, HbA1c, Blood glucose level, and BMI
3	Polyuria, Polydipsia, Gender, Age, Partial paresis, Irritability, and Genital thrush
4	Glucose, Insulin, BMI, Age, and Pregnancies

### 5.6 Metrics before feature selection

The accuracy, precision, recall, and F1-score for different classifiers applied to the four datasets are documented in Table 8. These results reveal that the Random Forest classifier surpasses others on Dataset-1, exhibiting the highest values in accuracy, recall, and F1-score. Following the Random Forest, the XGBClassifier demonstrates marginally lower performance, while the Gradient Boosting Classifier ranks third in terms of accuracy.

**Table 8:** Classification metrics without feature selection for datasets 1-4.

**Dataset**	**Models**	**Accuracy**	**Precision**	**Recall**	**F1-Score**
Dataset-1	XGBClassifier	0.937	0.937	0.937	0.952
	RandomForestClassifier	0.942	0.942	0.942	0.956
	GradientBoostingClassifier	0.932	0.932	0.932	0.948
	Support Vector Machine	0.832	0.830	0.832	0.874
Dataset-2	XGBClassifier	0.923	0.921	0.923	0.768
	RandomForestClassifier	0.933	0.936	0.933	0.778
	GradientBoostingClassifier	0.937	0.937	0.937	0.796
	Support Vector Machine	0.910	0.907	0.910	0.703
Dataset-3	XGBClassifier	0.971	0.974	0.971	0.978
	RandomForestClassifier	0.990	0.991	0.990	0.993
	GradientBoostingClassifier	0.971	0.974	0.971	0.978
	Support Vector Machine	0.894	0.895	0.894	0.922
Dataset-4	XGBClassifier	0.982	0.982	0.982	0.973
	RandomForestClassifier	0.982	0.982	0.982	0.973
	GradientBoostingClassifier	0.881	0.880	0.881	0.813
	Support Vector Machine	0.780	0.775	0.780	0.623

Regarding Datset-2, the Gradient Boosting classifier achieves the highest accuracy, precision, recall, and F1-score, followed by the Random Forest classifier, XGB classifier, and SVM. Besides, the Random Forest classifier outperforms others on the Datset-3, achieving the highest values in terms of accuracy, recall, and F1-score. Furthermore, the Gradient Boosting Classifier and the XGB Classifier demonstrate comparable performance. Additionally, both the Random Forest classifier and XGB Classifier surpass the other classifiers on Dataset-4, exhibiting almost equal and the highest values in terms of accuracy, recall, and F1-score. In contrast, the Gradient Boosting Classifier and the SVM have the lowest values in terms of these metrics.

### 5.7 Performance evaluation after feature selection

The performance evaluation results after using feature selection are depicted in Table 9. For instance, regarding Dataset-1, the selected features included Regular Medicine, Age, BMI, Sound Sleep, BP Level, Stress, and physical activity. Through various classification algorithms, it was determined that the highest accuracy was achieved using both the random forest classifier and the XGBoost classifier. However, it is noteworthy that the overall accuracy, when utilizing important features, is notably lower compared to the accuracy of classifiers that do not utilize these crucial features. Moving forward, for Dataset-2, the features selected for analysis were age, hypertension, HbA1c, blood glucose level, and BMI. The random forest classifier demonstrated the highest accuracy among the tested algorithms. Moreover, the accuracy of the model after feature selection was greater than the accuracy achieved prior to feature selection. Additionally, on Dataset-3. Typically, the selected features included Polyuria, Polydipsia, Gender, Age, partial paresis, Irritability, and Genital thrush. The highest accuracy was achieved using the XGBoost classifier. It was also noted that while the highest accuracy after feature selection remained the same as before feature selection, some models exhibited increased accuracy following feature selection. Lastly, based on the performance obtained on Dataset-4, the features selected for this dataset are glucose, insulin, BMI, age, and pregnancies. The random forest classifier achieved the highest accuracy among the models tested. Furthermore, the highest accuracy after feature selection was greater than the accuracy before feature selection.

**Table 9:** Performance obtained on Datasets 1-4 after feature selection.

**Dataset**	**Models**	**Accuracy**	**Precision**	**Recall**	**F1-Score**
Dataset-1	XGBClassifier	0.937	0.937	0.937	0.952
	RandomForestClassifier	0.937	0.937	0.937	0.952
	GradientBoostingClassifier	0.932	0.932	0.932	0.948
	Support Vector Machine	0.801	0.811	0.801	0.840
Dataset-2	XGBClassifier	0.933	0.933	0.933	0.787
	RandomForestClassifier	0.940	0.942	0.940	0.804
	GradientBoostingClassifier	0.937	0.937	0.937	0.796
	Support Vector Machine	0.913	0.912	0.913	0.711
Dataset-3	XGBClassifier	0.990	0.991	0.990	0.993
	RandomForestClassifier	0.990	0.991	0.990	0.993
	GradientBoostingClassifier	0.981	0.982	0.981	0.986
	Support Vector Machine	0.923	0.924	0.923	0.945
Dataset-4	XGBClassifier	0.982	0.982	0.982	0.973
	RandomForestClassifier	0.986	0.986	0.986	0.978
	GradientBoostingClassifier	0.847	0.845	0.847	0.762
	Support Vector Machine	0.767	0.760	0.767	0.610

#### 5.7.1 Performance enhancement

While feature selection did not universally enhance performance in this study, it still holds potential benefits for reducing model complexity and improving interpretability. By exploring alternative methods, fine-tuning hyperparameters, and incorporating domain knowledge, it is possible to achieve better performance and more efficient models.

- It is observed that the feature selection process did not consistently enhance the model's performance across all datasets. This outcome can be attributed to various factors, such as the intrinsic characteristics of the datasets and the specific feature selection methods employed.
- Feature selection is generally expected to improve model performance by eliminating irrelevant or redundant features. However, in some cases, it might lead to the loss of valuable information, which could negatively impact the model's accuracy.

#### 5.7.2 Model complexity

Although the feature selection process did not universally improve performance, it is crucial to acknowledge its potential impact on model complexity. By reducing the number of features, the complexity of the model can be decreased, which may result in:

- Faster training and prediction times.
- Reduced risk of overfitting, particularly in cases where the dataset is small or the number of features is large.
- Simplified interpretation of the model, making it easier for domain experts to understand the contributing factors to the model's predictions.

#### 5.7.3 Suggestions for improvement

- **Alternative feature selection methods:** It may be beneficial to explore different feature selection techniques, such as Recursive Feature Elimination (RFE), Principal Component Analysis (PCA), or other dimensionality reduction methods. These approaches could potentially yield a more optimal set of features, enhancing model performance and reducing complexity.
- **Ensemble methods:** Employing ensemble methods that combine multiple feature selection techniques could lead to a more robust feature set. This approach may help in retaining important features while eliminating redundant ones.
- **Hyperparameter tuning:** Fine-tuning the hyperparameters of the feature selection algorithms and the classifiers could improve the overall performance. Grid search or random search methods can be utilized to identify the best hyperparameter settings.
- **Cross-validation:** Implementing cross-validation techniques can provide a more reliable assessment of the model's performance and generalizability. This approach helps in ensuring that the results are not biased due to a particular train-test split.
- **Domain knowledge:** Incorporating domain knowledge into the feature selection process can be valuable. Consulting with domain experts to identify the most relevant features based on their expertise can enhance the model's performance and interpretability.

### 5.8 Ensemble approach

The stacking ensemble approach, utilizing four base models and a Logistic Regression meta-model, is applied to four datasets, with the results systematically tabulated in Table 10. The findings indicate that the performance of the stacking ensemble approach exceeds the conventional metrics for each dataset. However, its performance is inferior to that achieved using the feature selection method.

**Table 10:** Performance using stacking ensemble approach.

**Dataset**	**Accuracy**	**Precision**	**Recall**	**F1-Score**
Dataset-1	0.963	0.963	0.963	0.940
Dataset-2	0.933	0.933	0.933	0.787
Dataset-3	0.981	0.982	0.981	0.986
Dataset-4	0.982	0.982	0.982	0.973

### 5.9 Explainable AI

**Figure 4:**
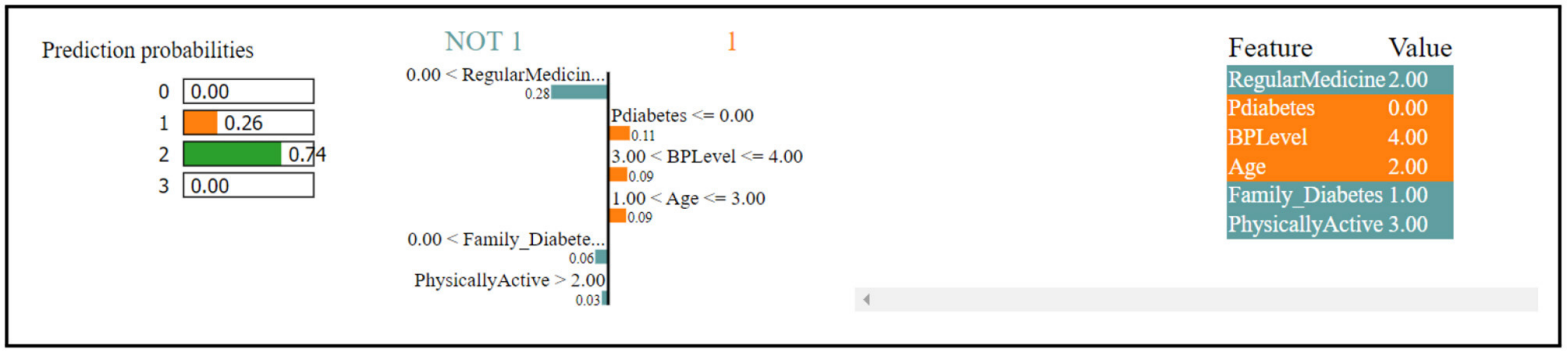
Dataset-1 lime.

Figure 4 indicates that the thirteenth record in the dataset has been predicted to have diabetes with a 74% confidence level. This prediction is primarily influenced by factors such as regular medication, family history of diabetes, and physical activity, which collectively contribute to the high confidence in the diabetes diagnosis. Contrastingly, the same record has been predicted to not have diabetes, albeit with a lower confidence of 26%. This opposing prediction is influenced by the values of BP level (blood pressure level) and age, which seem to indicate a lower risk of diabetes. In summary, the prediction for the thirteenth record tends toward a diabetes diagnosis due to certain features, while other features suggest the absence of diabetes, leading to a confidence level of 74% for diabetes and 26% for non-diabetes.

**Figure 5:**
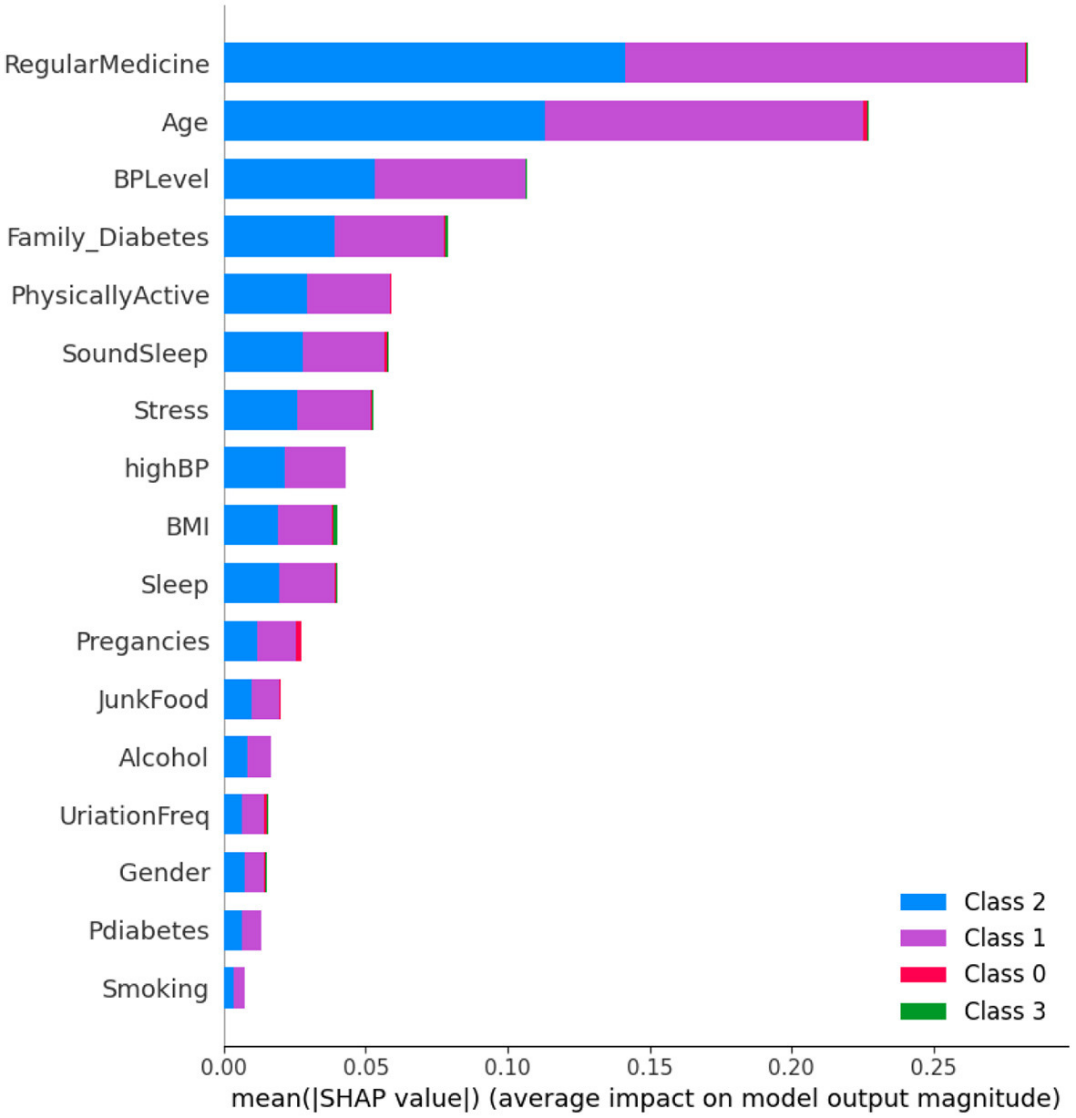
Dataset-1 SHAP feature importance plot.

The SHAP method was employed to analyze Dataset 1, with the results presented in the form of a figure. In Figure 5, class 1 denotes non-diabetic patients, while class 2 represents diabetic patients. It is evident that most features exhibit equal importance for both classes, indicating a balanced impact on the classification of both diabetes and non-diabetes.

However, a detailed analysis reveals that two factors, regular medication and age, play a significant role in the classification process. These features are crucial in determining the diabetic or non-diabetic status of a patient. Conversely, features such as prediabetes and smoking have minimal influence on the classification, suggesting they contribute less to the differentiation between the two groups.

**Figure 6:**
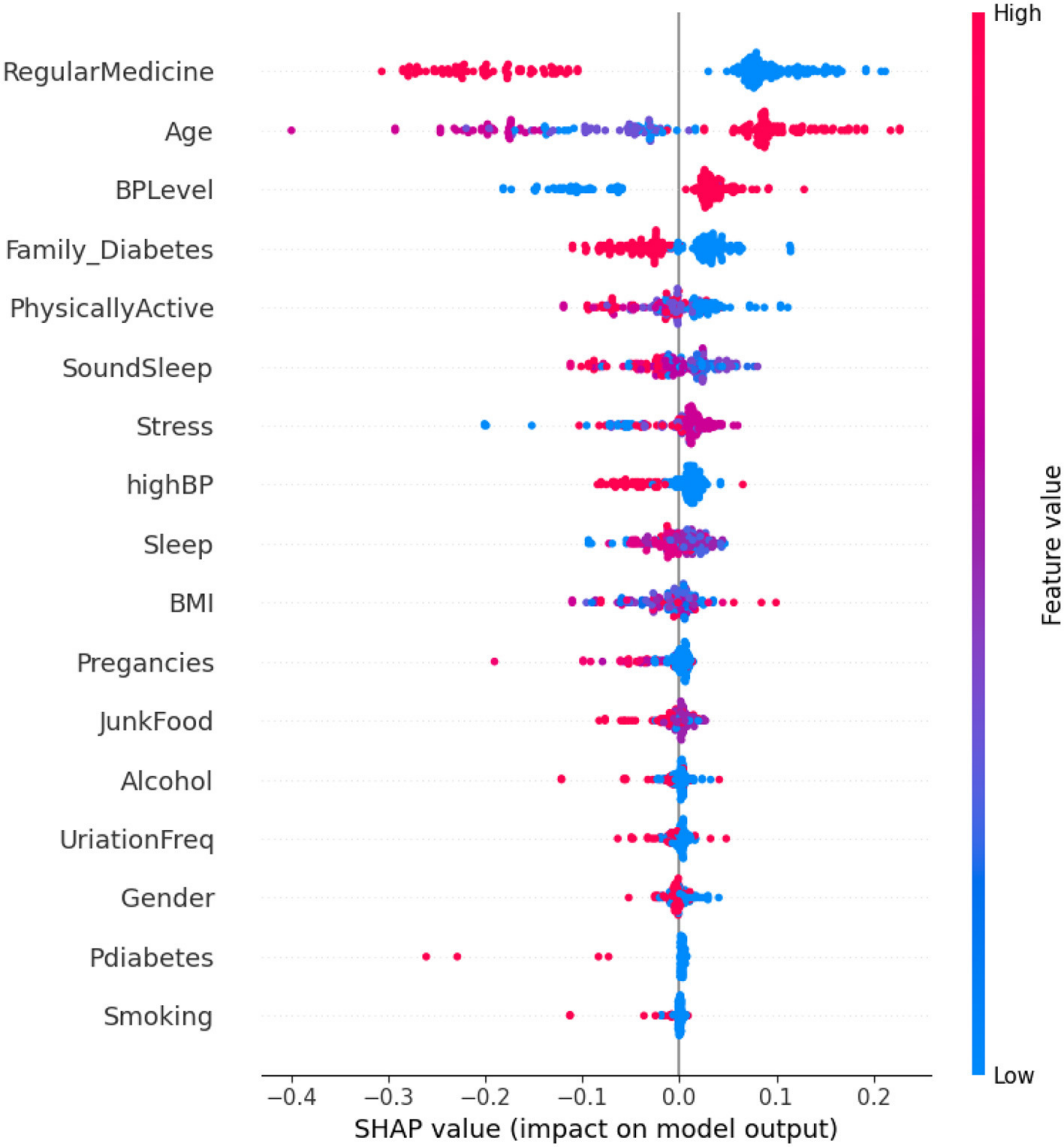
Dataset-1 SHAP summary plot.

Analyzing Figure 6, it is observed that individuals who adhere to a high regimen of regular medication tend to be non-diabetic. This suggests an association between the use of regular medication and a reduced risk of diabetes. Additionally, diabetes is more prevalent among elderly individuals as compared to younger ones, indicating that age is a significant factor in predicting diabetes, with older individuals being more susceptible. The widespread use of regular medication, combined with the influence of age, underscores the importance of these factors in predicting diabetes. In contrast, features such as diabetes and smoking appear to have negligible or no impact on the prediction of diabetes.

**Figure 7:**
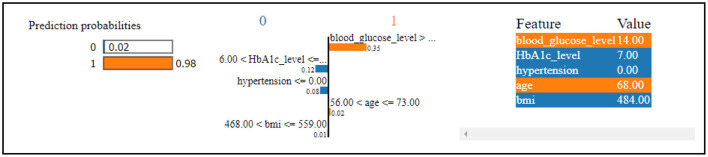
Dataset-2 lime explainer.

Figure 7 reveals that the thirteenth record in the data has been predicted to have diabetes with a 98% confidence level. This high confidence in the diabetes prediction is primarily influenced by the values of blood glucose level and age. Conversely, the same record has been predicted to not have diabetes, but with a considerably lower confidence of 2%. This diminished confidence in the absence of diabetes is attributed to the values of HbA1c level, hypertension, and BMI. The SHAP method was utilized for analyzing Dataset 2, with the findings presented graphically.

**Figure 8:**
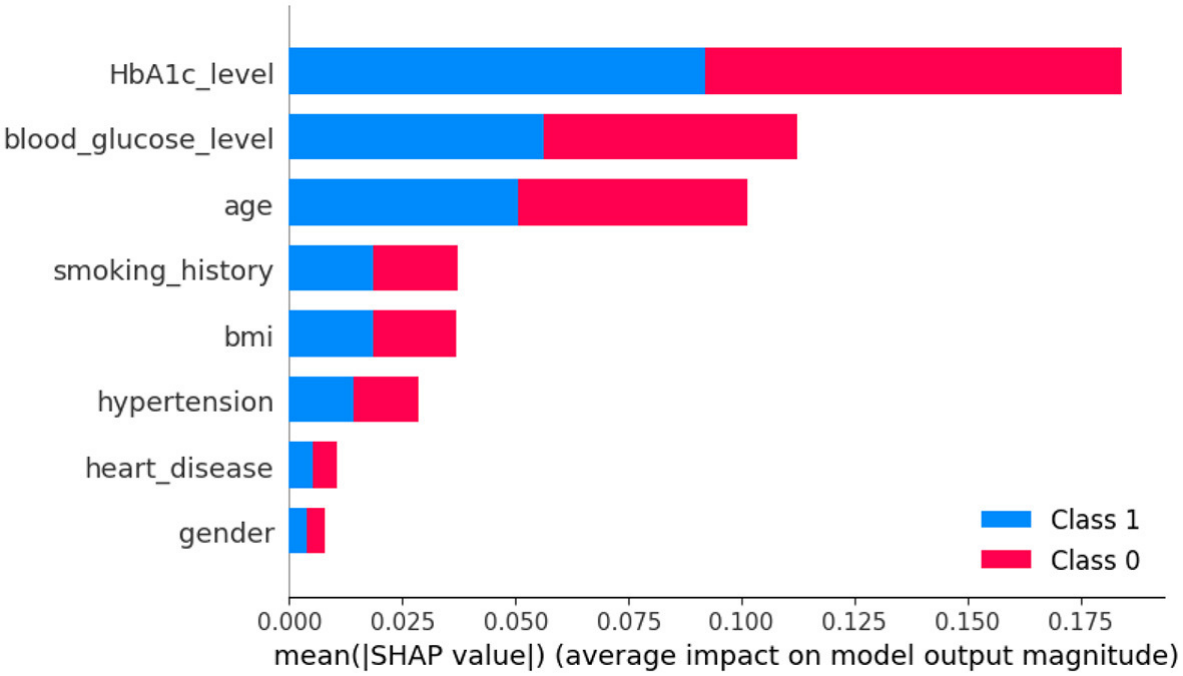
Dataset-2 SHAP feature importance plot.

In Figure 8, class 0 denotes non-diabetic patients, while class 1 represents diabetic patients. Notably, most features show similar contributions for both classes, implying an equally significant impact on the classification of individuals as diabetic or non-diabetic.

A deeper examination of the specifics reveals that three factors-HbA1c level, blood glucose level, and age-play pivotal roles in the classification process. These features exert a pronounced influence on determining a patient's diabetic status. Conversely, features such as heart disease and gender have a minimal impact on classification, suggesting they are less significant in distinguishing between the two groups.

**Figure 9:**
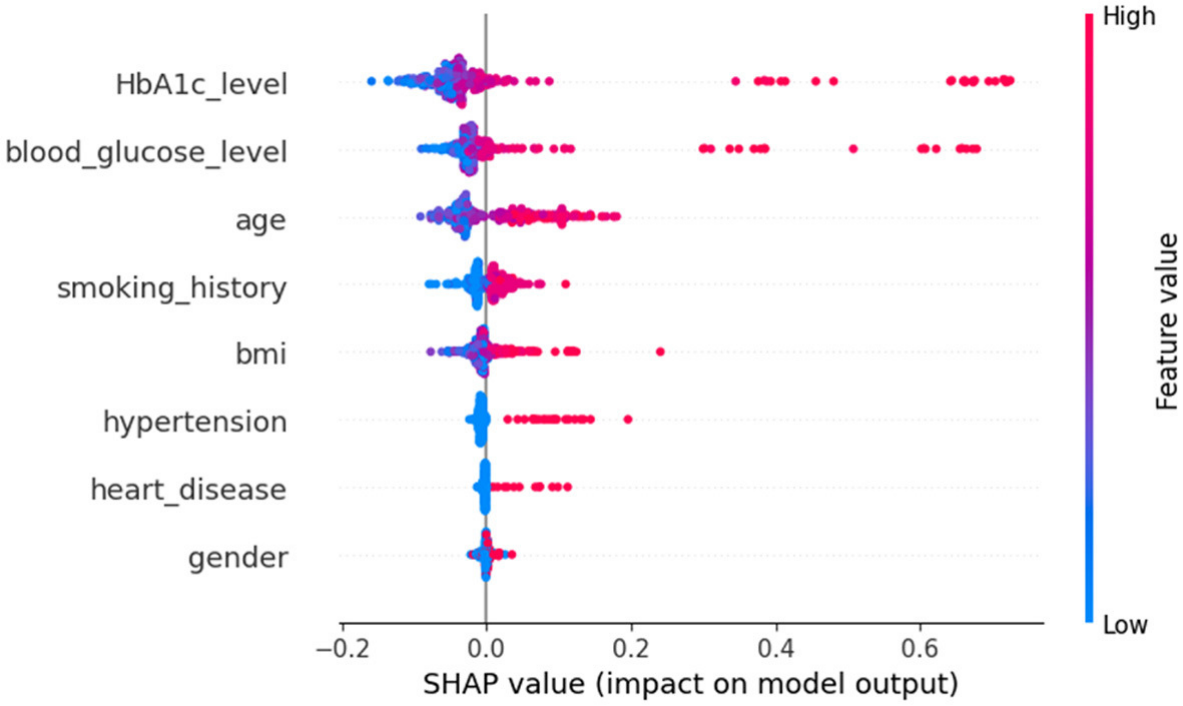
Dataset-2 SHAP summary plot.

Upon examining Figure 9, it becomes evident that individuals with elevated HbA1c levels are more likely to be diagnosed with diabetes. Similarly, high blood glucose levels are commonly associated with diabetes. This observation implies that both HbA1c level and blood glucose level are crucial factors in predicting diabetes. The frequent occurrence of high levels of HbA1c and blood glucose underscores their importance in making accurate predictions about diabetes. Conversely, features such as heart disease and gender seem to have minimal to no impact on the prediction of diabetes, indicating their limited contribution to differentiating between diabetic and non-diabetic individuals.

**Figure 10:**
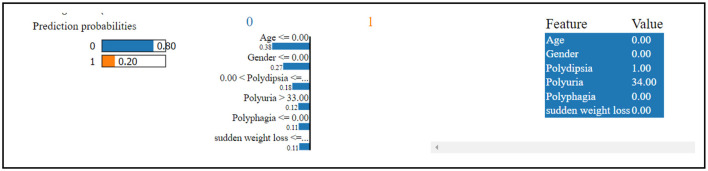
Dataset-3 lime explainer.

**Figure 11:**
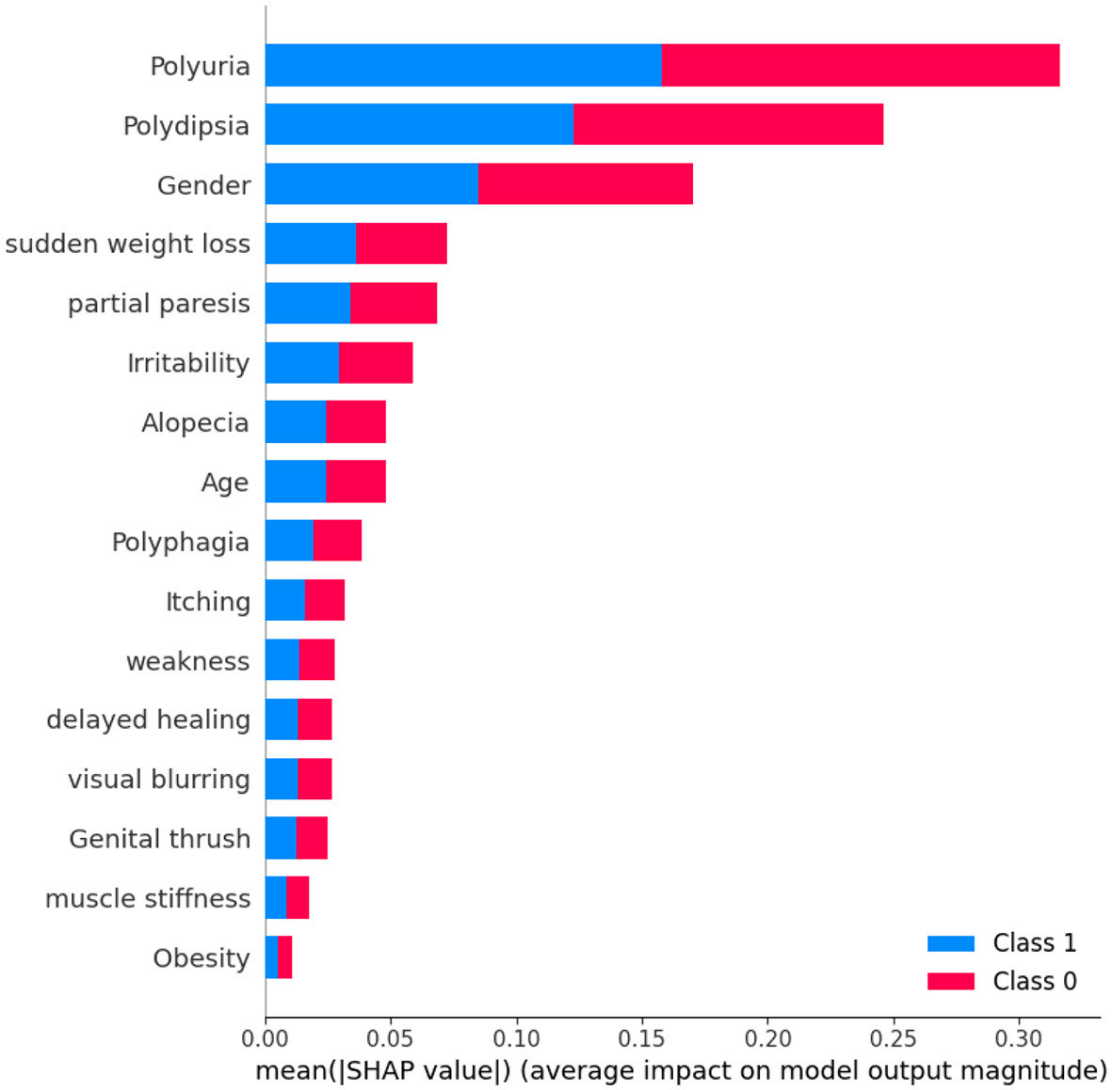
Dataset-3 SHAP feature importance plot.

Figure 10 shows that the thirteenth record in the dataset has been predicted to have diabetes with an 80% confidence level. This confident prediction of diabetes is primarily based on the values of gender, polydipsia, polyuria, and age. Conversely, the same record has been predicted to not have diabetes, albeit with a lower confidence of 20%. The SHAP method was employed to analyze Dataset 3, with the results depicted in a figure. In Figure 11, class 0 represents non-diabetic patients, while class 1 corresponds to diabetic patients. Notably, most features demonstrate similar contributions for both classes, indicating an equal impact on classifying individuals as diabetic or non-diabetic.

A detailed examination reveals that three factors-polyuria, polydipsia, and gender-play significant roles in the classification process. These features are pivotal in determining a patient's diabetic status. In contrast, features such as muscle stiffness and obesity appear to exert minimal influence on classification, suggesting they are less critical in distinguishing between the two groups.

**Figure 12:**
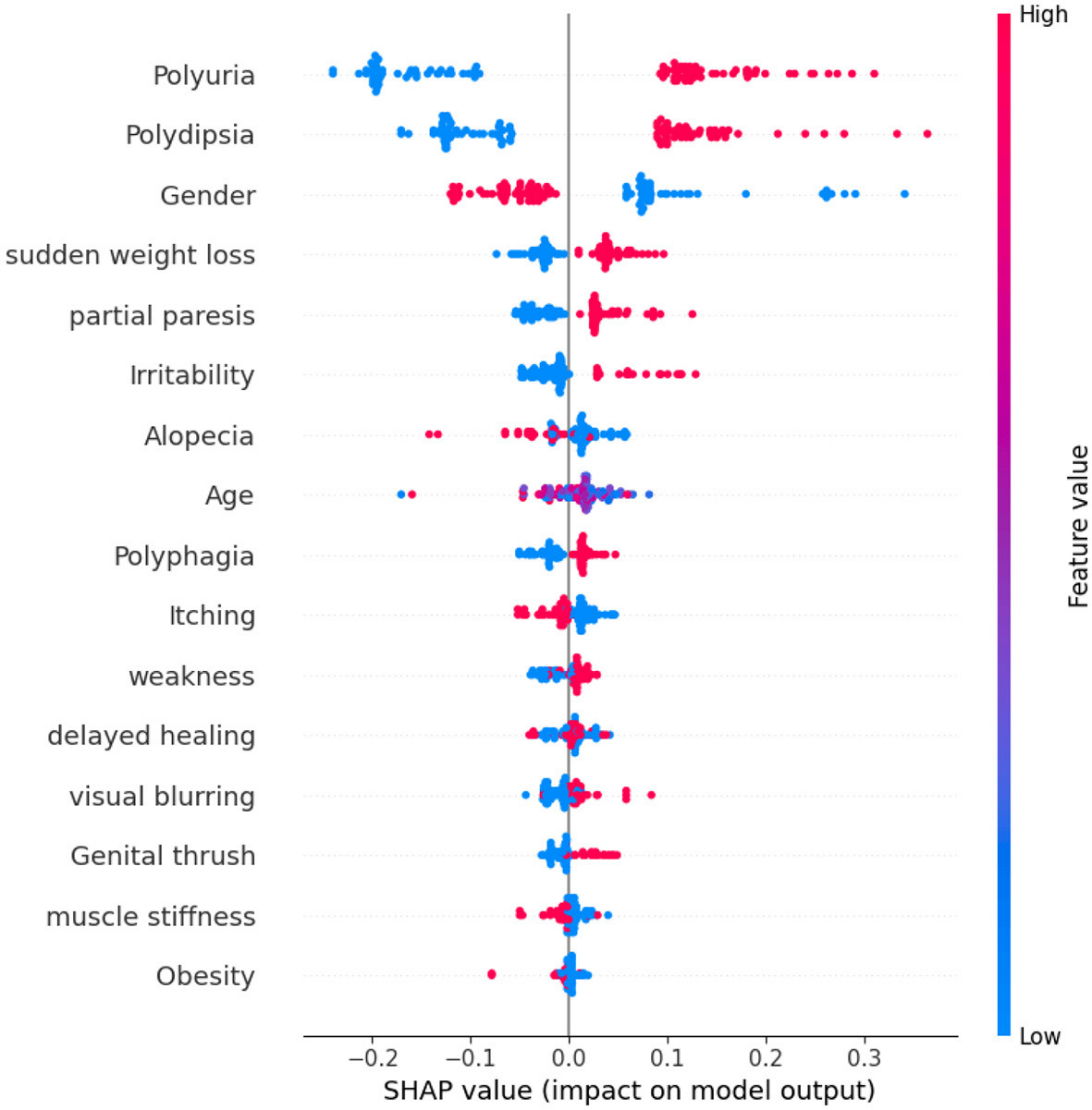
Dataset-3 SHAP summary plot.

Upon analyzing Figure 12, it becomes evident that individuals with high levels of polyuria are more likely to be diagnosed with diabetes. Similarly, the presence of polydipsia is commonly associated with the condition. This observation indicates that both polyuria and polydipsia are crucial factors in predicting diabetes. The frequent occurrence of these symptoms underscores their importance in making accurate predictions about diabetes. On the other hand, features such as muscle stiffness and obesity seem to have minimal to no impact on the prediction of diabetes, suggesting that they play a less significant role in differentiating between diabetic and non-diabetic individuals.

**Figure 13:**
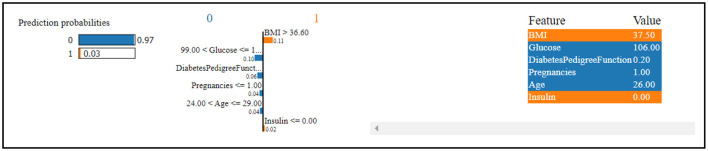
Dataset-4 lime explainer.

Figure 13 indicates that the thirteenth record in the dataset has been predicted to have diabetes with a high confidence level of 97%. This strong prediction of diabetes is primarily influenced by the values of glucose level, Diabetes Pedigree Function, pregnancies, and age.

In contrast, the same record has been predicted to not have diabetes, albeit with a low confidence level of 3%. This less confident prediction is attributed to the values of insulin and BMI (Body Mass Index).

**Figure 14:**
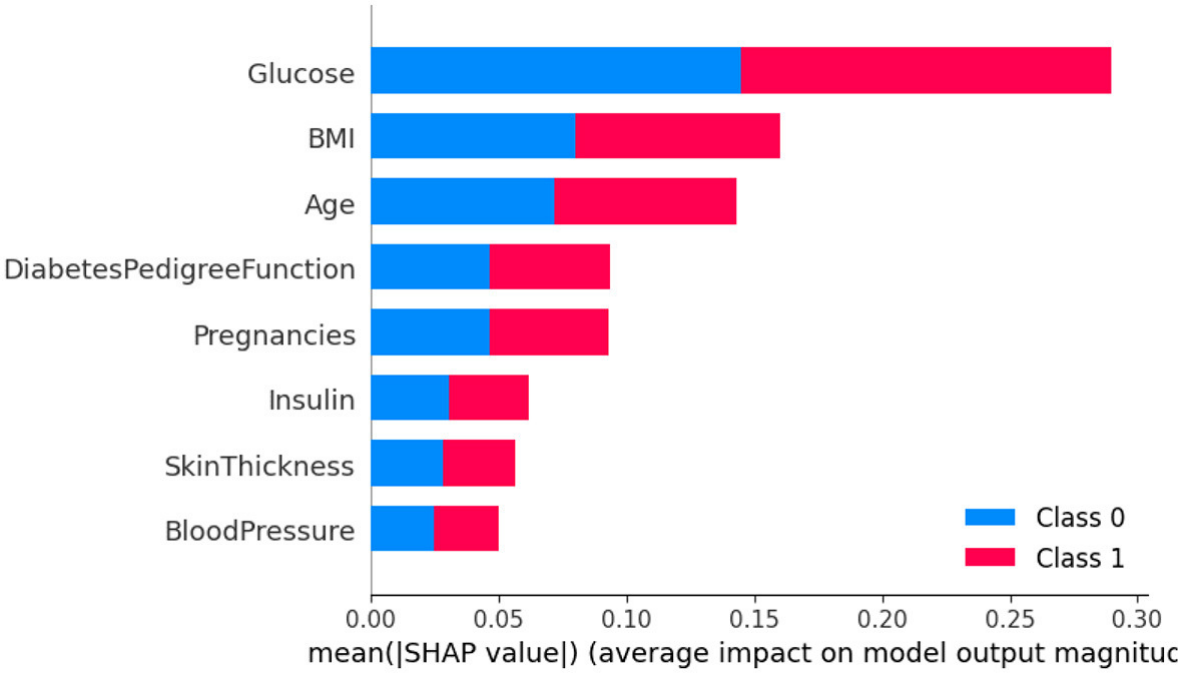
Dataset-4 SHAP feature importance plot.

The SHAP method was employed to analyze Dataset 4, with the results being presented graphically in Figure 14. In this figure, class 0 is indicative of non-diabetic patients, while class 1 denotes diabetic patients. It is apparent from the analysis that most features contribute similarly for both classes, suggesting an equal impact on classifying individuals as either diabetic or non-diabetic.

Further scrutiny of the specific details reveals that three factors-glucose level, BMI (Body Mass Index), and age-hold pivotal roles in the classification process. These features are critical in determining a patient's diabetic status. In contrast, features such as skin thickness and blood pressure appear to exert minimal influence on the classification, indicating their lesser contribution in differentiating between diabetic and non-diabetic individuals.

**Figure 15:**
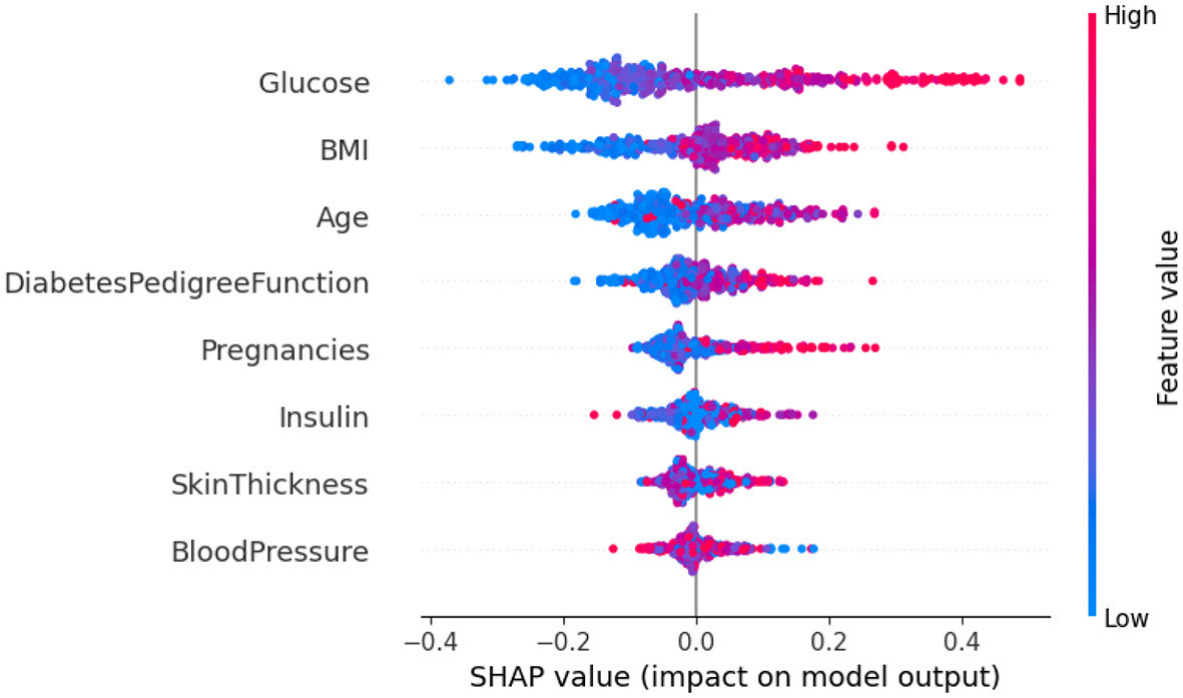
Dataset-4 SHAP summary plot.

Upon analyzing Figure 15, it becomes clear that individuals with high glucose levels are more likely to be diagnosed with diabetes. Similarly, a high BMI (Body Mass Index) is often associated with the condition. This observation indicates that both glucose level and BMI are crucial factors in predicting diabetes. The frequent occurrence of high glucose levels and high BMI underscores their importance in making accurate predictions about diabetes. In contrast, features such as skin thickness and blood pressure seem to have minimal to no impact on the prediction of diabetes, suggesting their limited role in differentiating between diabetic and non-diabetic individuals.

## 6 Conclusion

The increasing prevalence of diabetes necessitates the development of accurate and reliable predictive models for early diagnosis and effective management of the disease. These models can identify individuals at risk, enabling timely interventions to prevent or delay the onset of diabetes-related complications. Furthermore, predictive models assist healthcare providers in personalizing treatment plans based on individual risk profiles, ultimately improving patient outcomes. In our research, similar to the findings of Walaa Hassan (Sheta et al., 2024), who identified “Pregnancies,” “Glucose,” “BMI,” “Pedigree Function,” and “Age” as important features, we determined that “Age,” “Glucose,” and “BMI” are among the most significant factors in predicting diabetes. Generally, the Random Forest classifier outperforms other classifiers in handling diabetes datasets. Similarly, classifiers employing wrapper-based methods typically achieve higher accuracy than those using filter-based methods, with notable exceptions. The model's accuracy tends to decrease when limited to only important features rather than utilizing all available features. Among the various wrapper-based methods, Gradient Boosting consistently delivers superior results, emerging as the top performer. Conversely, Fisher's Score is identified as the most effective among filter-based methods.

In this study, we implemented a stacking ensemble approach using four base models and a Logistic Regression meta-model across four datasets. The systematically tabulated results reveal that the performance of the stacking ensemble approach surpasses conventional metrics for each dataset, although it is slightly inferior to the feature selection method. Additionally, the integration of explainable AI techniques, such as Local Interpretable Model-agnostic Explanations (LIME) and SHapley Additive exPlanations (SHAP), provides valuable insights into the decision-making processes of the predictive models. These methods underscore the importance of features like age and Body Mass Index (BMI) as crucial factors in diabetes prediction. The inclusion of these explainable AI approaches enhances the transparency and interpretability of the models, highlighting the significance of specific features in improving diabetes prediction accuracy. This comprehensive approach illustrates the potential of combining ensemble methods with feature selection and explainable AI to develop robust predictive models for diabetes.

The study's proposed predictive models for diabetes diagnosis show limitations such as reduced generalizability due to dataset specificity, decreased accuracy when using only selected important features, and performance dependency on specific classifiers like Random Forest. Inconsistencies in feature importance across different datasets and methods, alongside challenges posed by integrating explainable AI techniques, reveal that some features minimally impact predictions, suggesting potential inefficiencies in model interpretation. Additionally, while ensemble methods like stacking enhance performance, they increase model complexity, which could complicate maintaining performance without overfitting. These limitations suggest areas for future refinement to enhance the models' robustness and applicability across diverse clinical settings.

## Data availability statement

Publicly available datasets were analyzed in this study. This data can be found here: https://www.kaggle.com/datasets/tigganeha4/diabetes-dataset-2019 (accessed February 12, 2024); https://www.kaggle.com/datasets/iammustafatz/diabetes-prediction-dataset (accessed February 12, 2024); https://www.kaggle.com/datasets/andrewmvd/early-diabetes-classification (accessed February 12, 2024); https://www.kaggle.com/datasets/mathchi/diabetes-data-set (accessed February 12, 2024).

## Author contributions

JK: Conceptualization, Data curation, Investigation, Methodology, Software, Writing – original draft. IJS: Data curation, Investigation, Methodology, Software, Writing – original draft, Formal analysis. SS: Data curation, Investigation, Software, Writing – original draft, Conceptualization. TA: Conceptualization, Data curation, Investigation, Software, Writing – original draft, Methodology. RRR: Methodology, Investigation, Software, Writing – original draft. YS: Formal analysis, Project administration, Supervision, Validation, Visualization, Writing – review & editing. DV-G: Writing – review & editing, Funding acquisition. YH: Writing – review & editing, Funding acquisition, Resources, Formal analysis, Project administration, Supervision, Visualization. WM: Funding acquisition, Project administration, Supervision, Validation, Visualization, Writing – review & editing. SA: Funding acquisition, Project administration, Resources, Supervision, Visualization, Writing – review & editing. KS: Formal analysis, Project administration, Supervision, Validation, Visualization, Writing – review & editing.
