# Supplementary material for: Analyzing classification and feature selection strategies for diabetes prediction across diverse diabetes datasets
Source: Front Artif Intell. 2024 Aug 21;7:1421751. doi: 10.3389/frai.2024.1421751 (PMC11371799; doi:10.3389/frai.2024.1421751)
Supplement: Supplementary file 1 [file Data_Sheet_1.docx]

**Supplementary Figures**


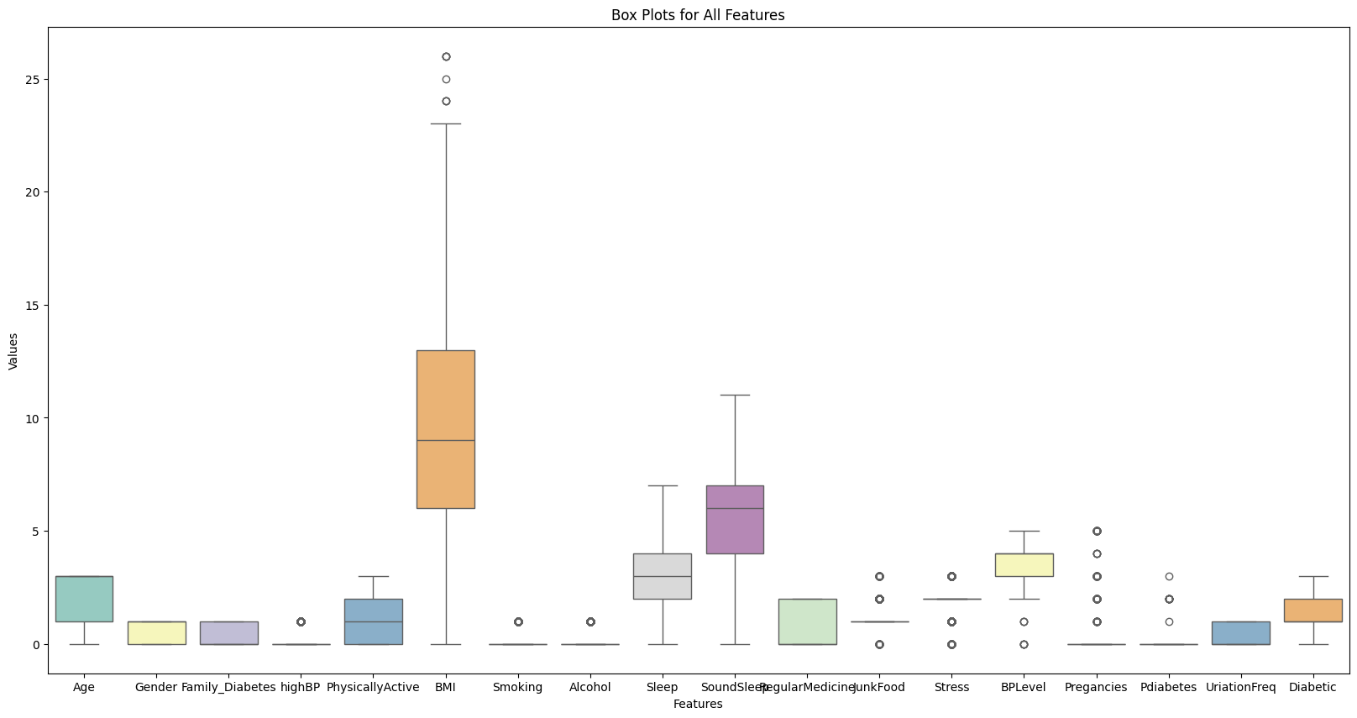


**Supplementary Figure 1.  Dataset-1 Features Boxplot**


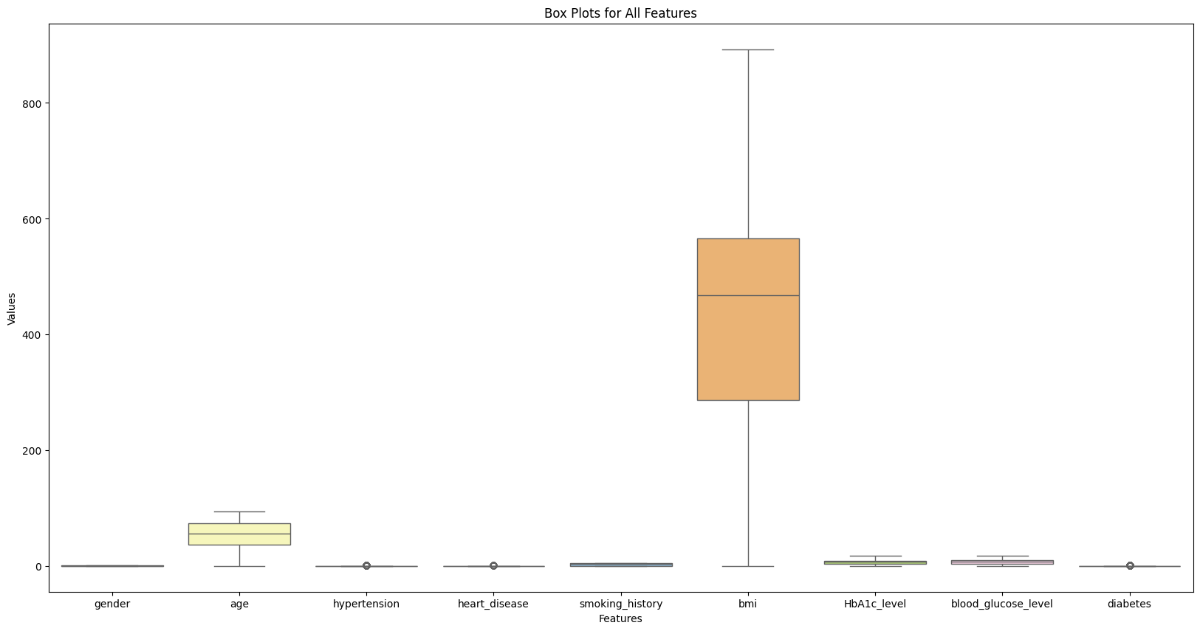


**Supplementary Figure 2.  Dataset-2 Features Boxplot**


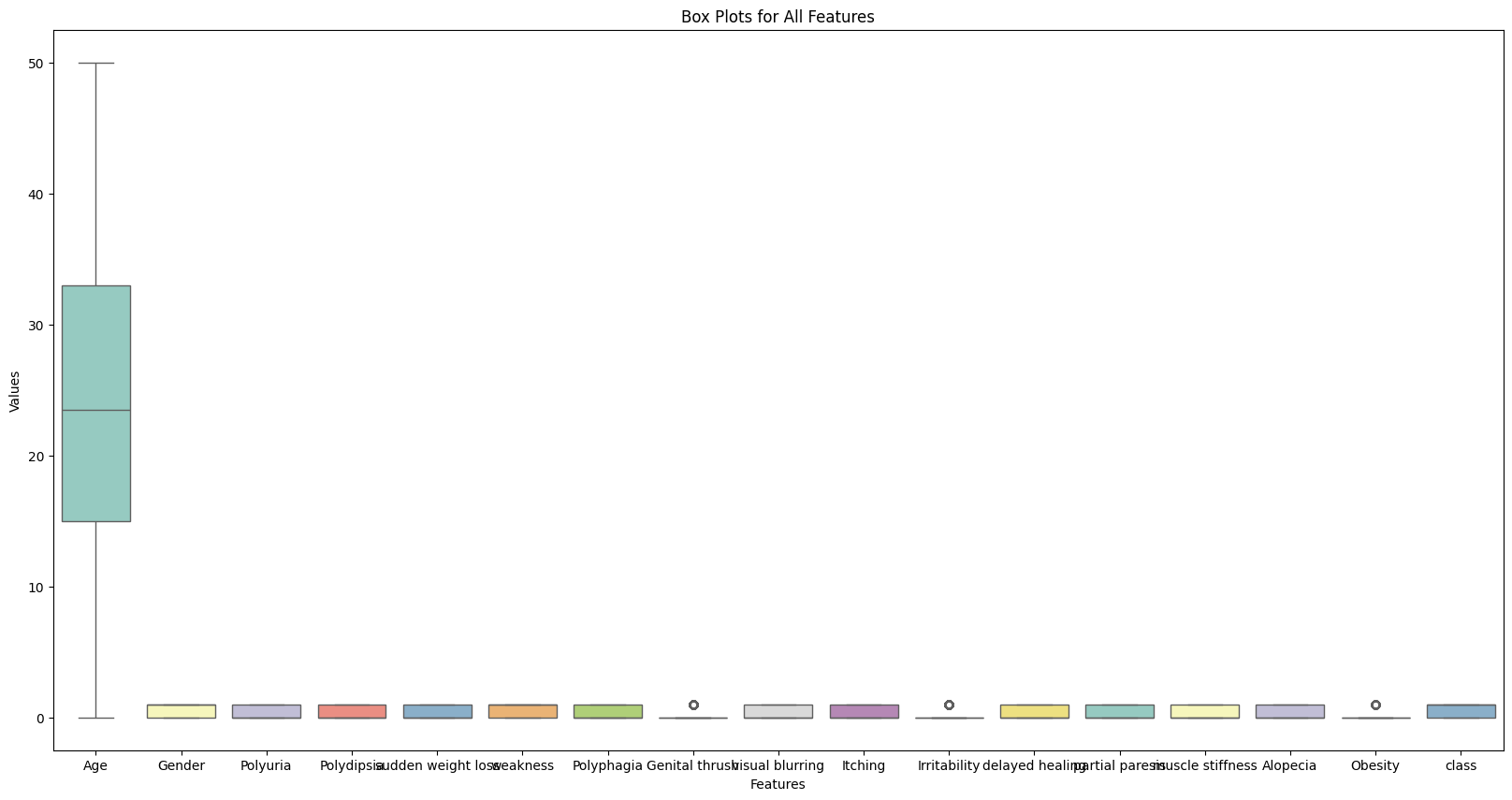


**Supplementary Figure 3.  Dataset-3 Features Boxplot.**


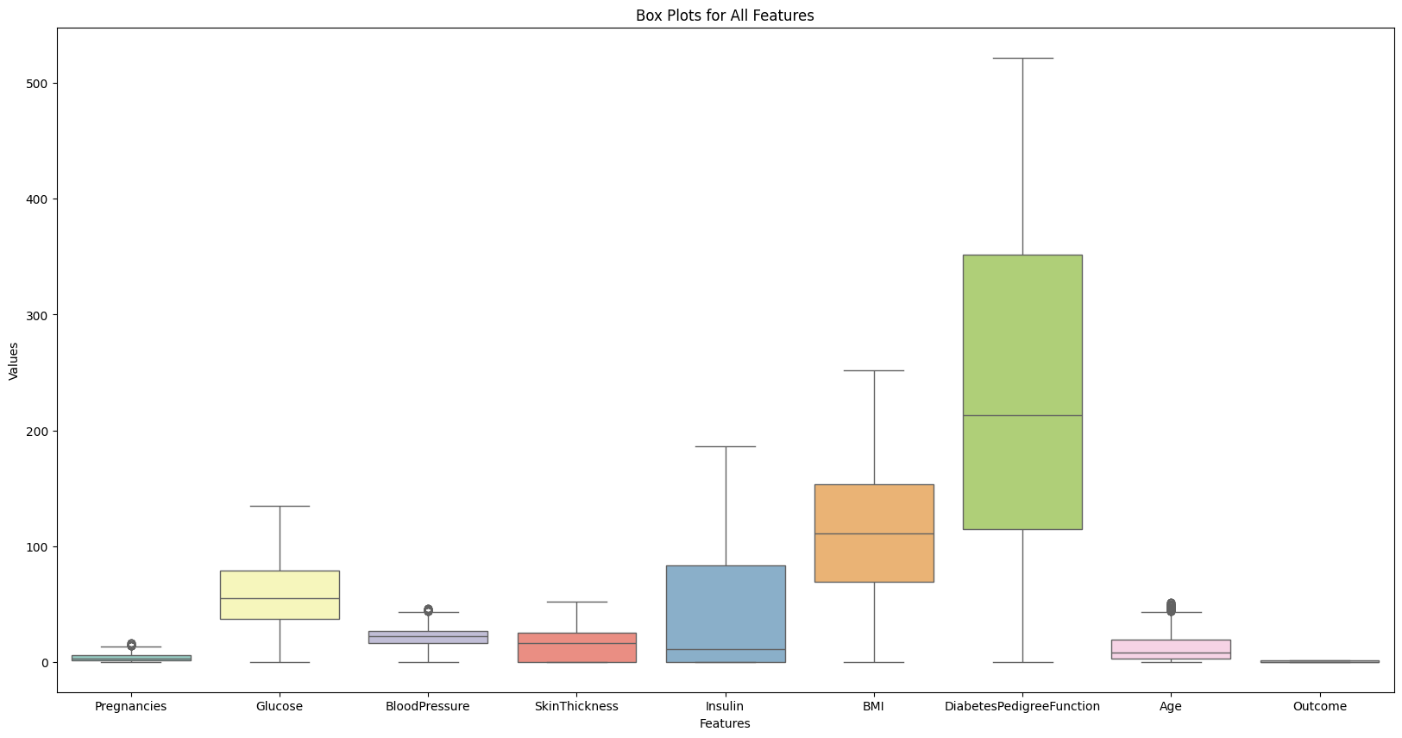


**Supplementary Figure 4**.  Dataset-4 Features Boxplot


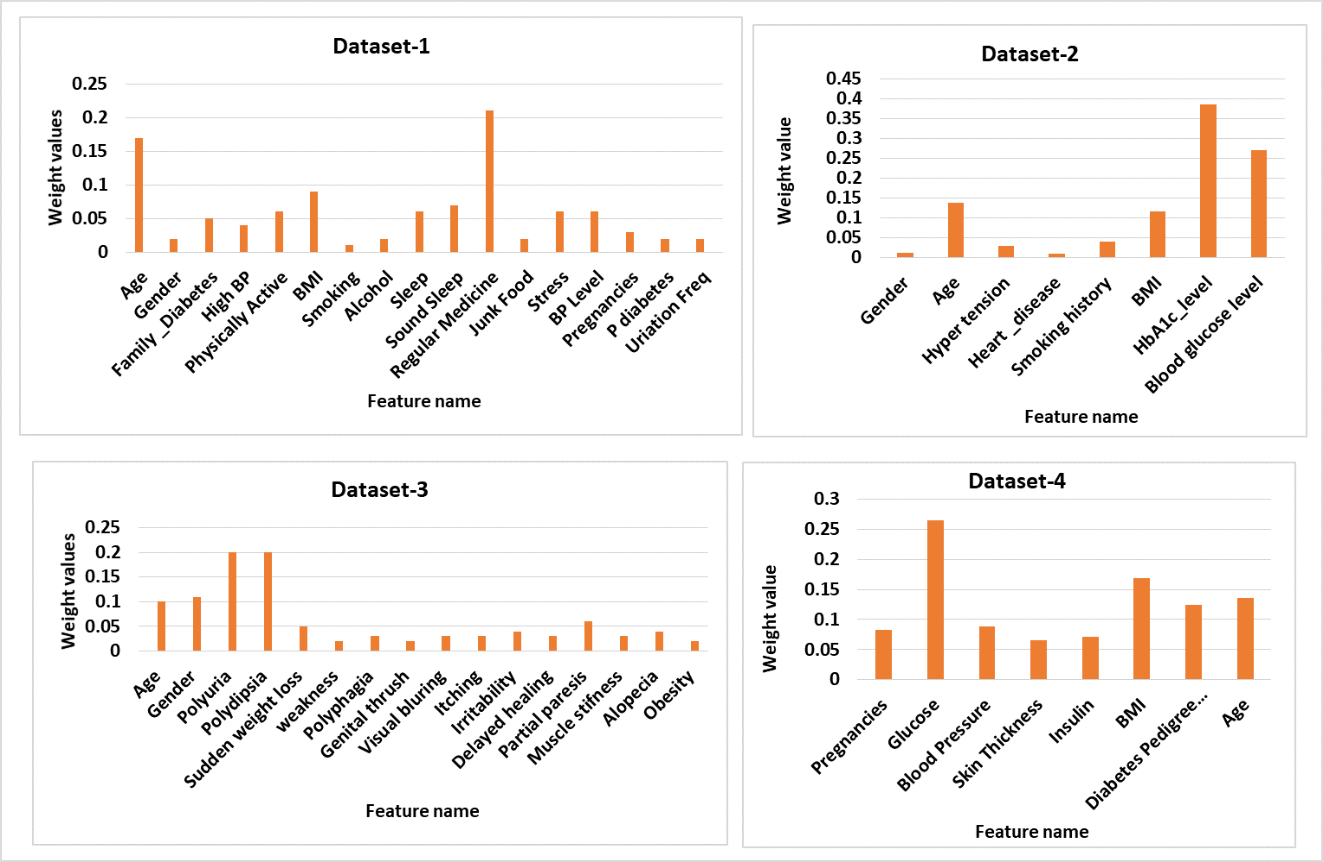


Supplementary Figure 5. Feature importance score using wrapper based approach with Random forest. Feature importance score for Dataset-1(top left); Feature importance score for Dataset-2, top right); Feature importance score for Dataset-3 (low left); Feature importance score for Dataset-4 (low right).


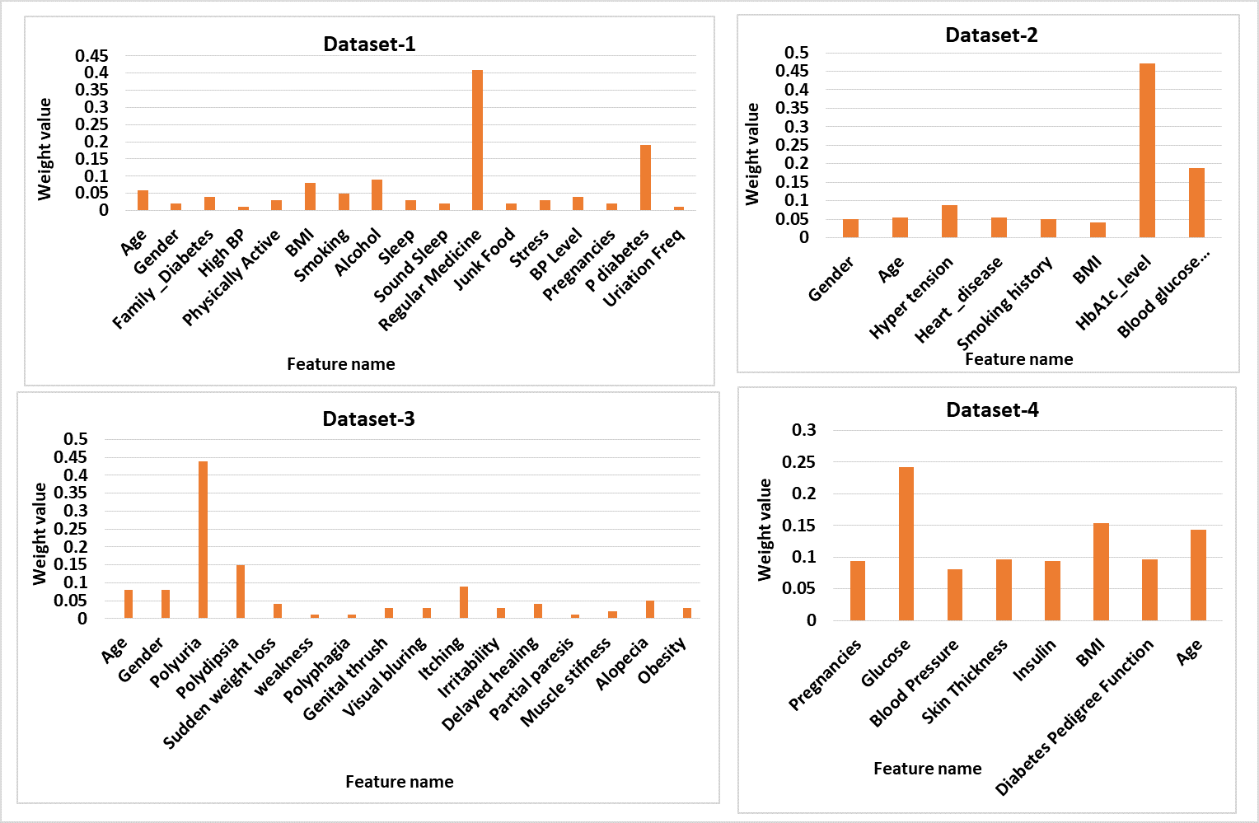


Supplementary Figure 6. Feature importance score using wrapper based approach with XGBoost. Feature importance score for Dataset-1(top left); Feature importance score for Dataset-2, top right); Feature importance score for Dataset-3 (low left); Feature importance score for Dataset-4 (low right).


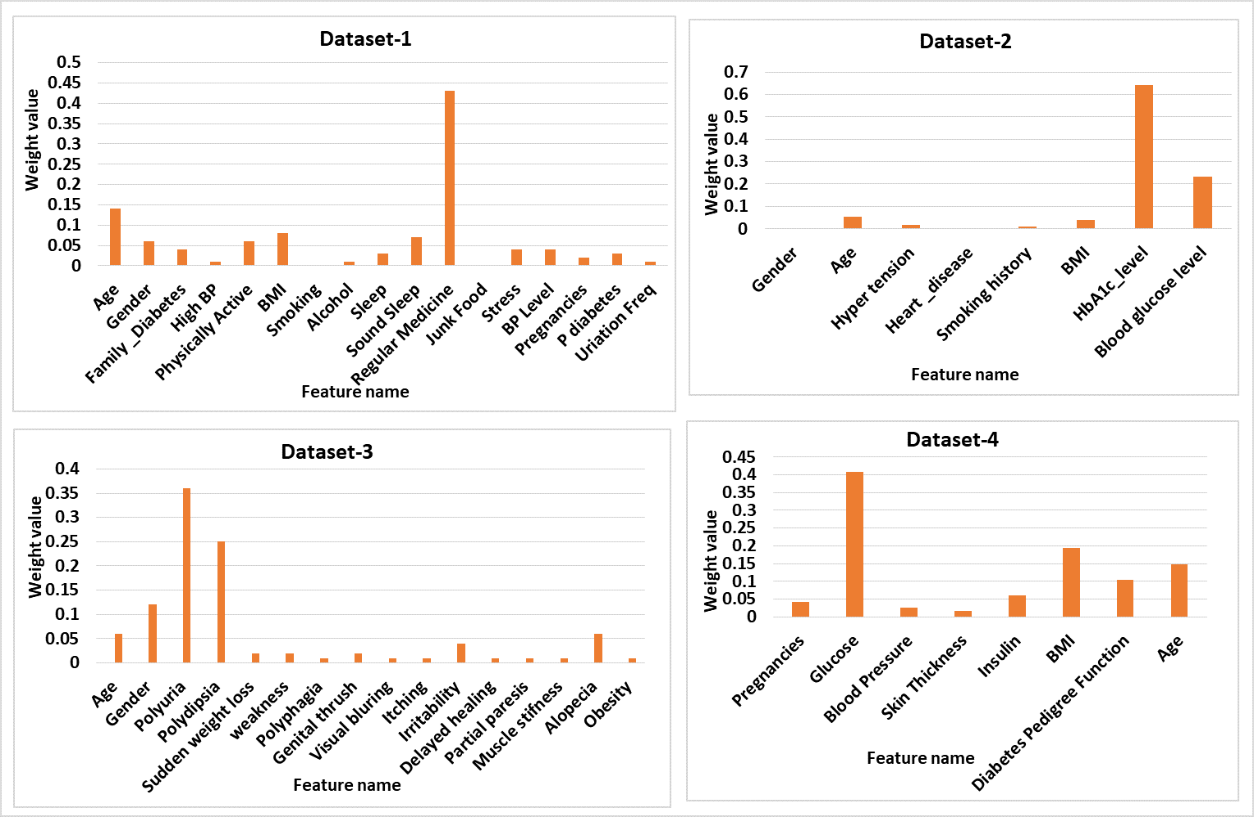


Supplementary Figure 7. Feature importance score using wrapper based approach with Gradient Boosting. Feature importance score for Dataset-1(top left); Feature importance score for Dataset-2, top right); Feature importance score for Dataset-3 (low left); Feature importance score for Dataset-4 (low right).


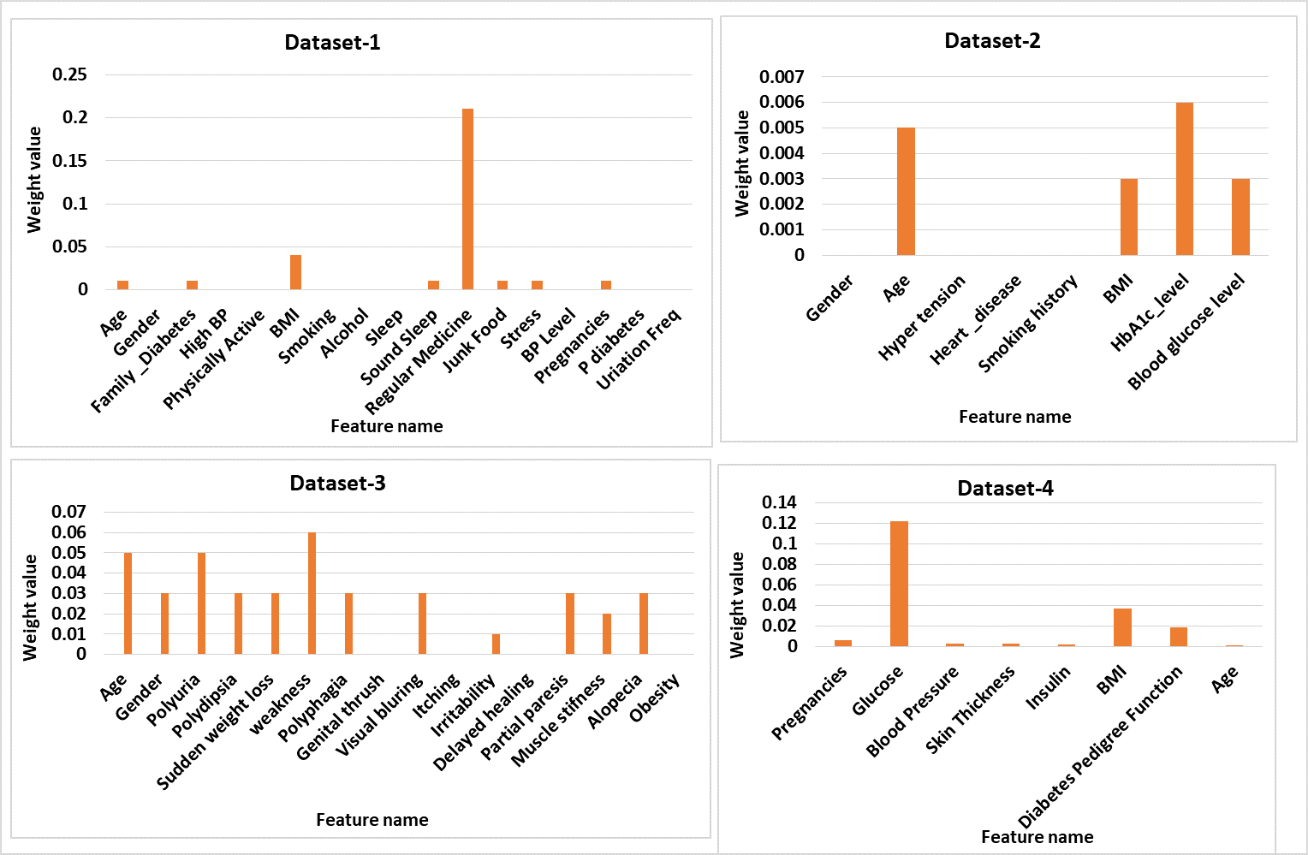


Supplementary Figure 8. Feature importance score using wrapper based approach with SVM. Feature importance score for Dataset-1(top left); Feature importance score for Dataset-2, top right); Feature importance score for Dataset-3 (low left); Feature importance score for Dataset-4 (low right).


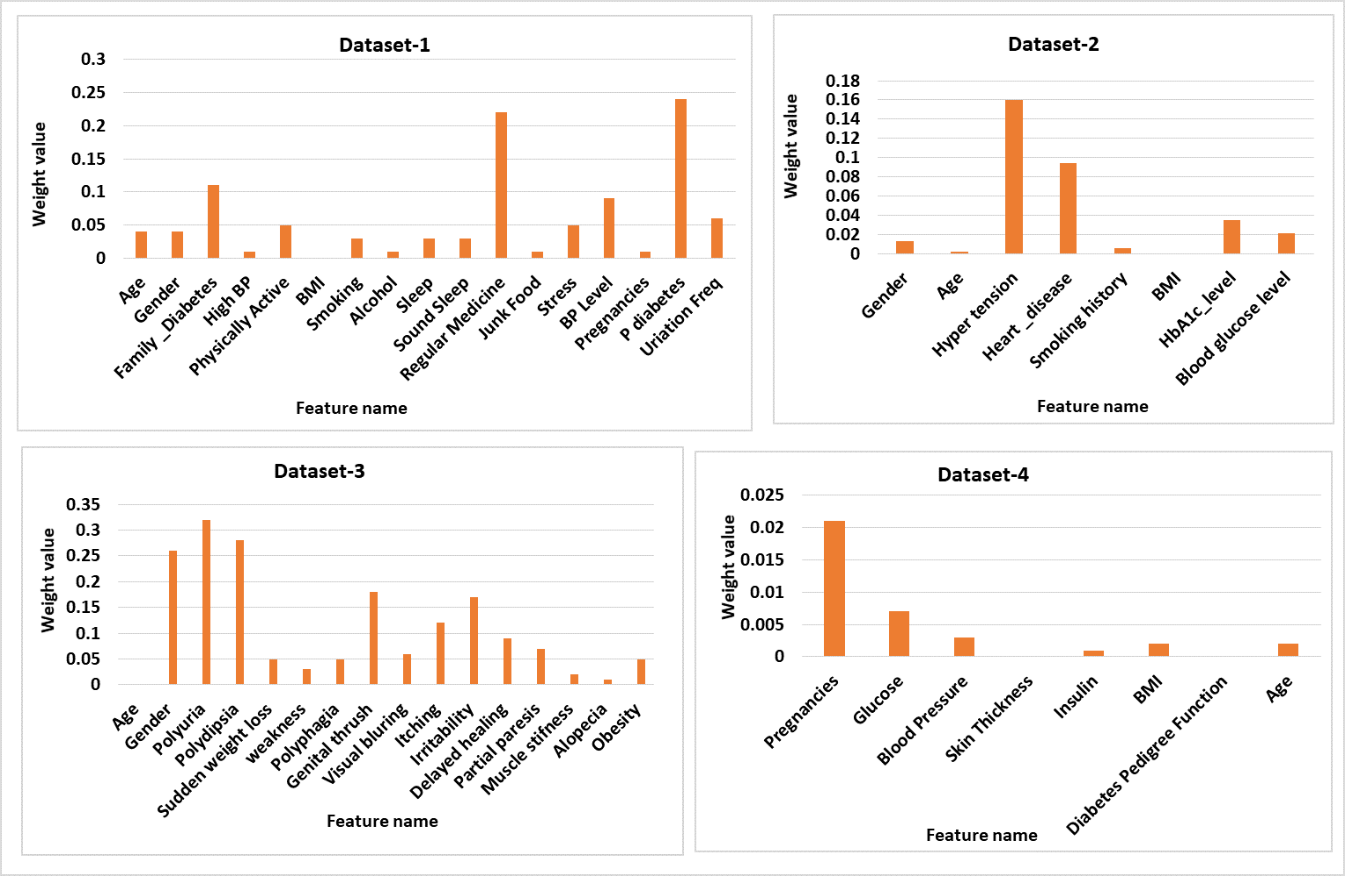


Supplementary Figure 9. Feature importance score using wrapper based approach with Linear Regression. Feature importance score for Dataset-1(top left); Feature importance score for Dataset-2, top right); Feature importance score for Dataset-3 (low left); Feature importance score for Dataset-4 (low right).
